# Physiological APP processing is required for synaptic plasticity and cognition

**DOI:** 10.1038/s41419-026-09378-6

**Published:** 2026-11-04

**Authors:** Lara Kilian, Verena Bengelsdorff, Jennifer Just, Sebastian Stork, Christos Galanis, Marija Banicevic, Irmgard Amrein, Michaela Back, Tassilo Jungenitz, Meike Fellenz, Jakob von Engelhardt, Thomas Deller, David P. Wolfer, Andreas Vlachos, Martin Korte, Ulrike C. Müller

**Affiliations:** 1 Department of Functional Genomics, Institute of Pharmacy and Molecular Biotechnology (IPMB), Ruprecht-Karls-Universität Heidelberg, Heidelberg, Germany; 2 Institute for Cell- and Neurobiology, TU Braunschweig, Braunschweig, Germany; 3 Department of Neuroanatomy, Institute of Anatomy and Cell Biology, University of Freiburg, Freiburg, Germany; 4 Department of Anatomy, University of Zürich, Zürich, Switzerland; 5 Institute of Human Movement Sciences and Sport, D-HEST, ETH Zürich, Zürich, Switzerland; 6 Focus Program Translational Neurosciences (FTN), University Medical Center of the Johannes Gutenberg University Mainz, Institute of Pathophysiology, Mainz, Germany; 7 Institute for Clinical Neuroanatomy, Goethe University Frankfurt, Frankfurt/Main, Germany; 8 Neuroinflammation and Neurodegeneration Group, Helmholtz Centre for Infection Research, Braunschweig, Germany

**Keywords:** Alzheimer's disease, Working memory, Molecular neuroscience, Spine plasticity, Long-term potentiation

## Abstract

While the accumulation of Aβ peptides, derived by proteolytic cleavage from the amyloid precursor protein APP, constitutes a hallmark of Alzheimer’s disease, the question of whether endogenous, physiological APP processing serves an essential role for normal brain function(s) has remained elusive. Here, we modified the mouse APP locus to delete the endogenous α- and β-secretase sites (APP residues S592-G621) while preserving the transmembrane topology and the APP C-terminus. These novel knockin mice (termed APPΔS622) lack APPsα, APPβ, and Aβ production and show only minimal soluble APPs shedding. These APPΔS622 mice provide a unique model to distinguish between functions mediated by unprocessed cell surface APP involved in synaptic adhesion or by proteolytic fragments. Strikingly, APPΔS622 mice exhibit pronounced impairments in both long-term potentiation (LTP) and homeostatic forms of synaptic plasticity. Extracellular field recordings revealed significant deficits in hippocampal LTP, associated with a reduced density of mature spines and reduced spine head size. LTP deficits could be restored by APPsα application, suggesting a requirement for non-amyloidogenic APP processing for LTP. In addition, APPΔS622 mice lacking endogenous Aβ displayed a lack of homeostatic synaptic scaling in response to network impairment that could be specifically rescued by exogenous Aβ. Importantly, these deficits in different forms of synaptic plasticity also led to impairments in cognitive behavior. In summary, our findings reveal an essential role of physiological APP processing products to differentially regulate complementary forms of synaptic plasticity that are critical for network stability, normal brain function and cognition.

## Introduction

Despite the molecular cloning of APP more than 30 years ago [1–3] and the subsequent genetic linkage of autosomal dominant APP mutations to familial forms of Alzheimer’s disease (AD) [4], the normal, physiological role of APP in the brain remains poorly understood. Strikingly, it is still unclear whether APP processing as such has an essential physiological role for brain function. In AD, synaptic dysfunction and loss of synapses occur early in the course of the disease, prior to overt Aβ accumulation. Importantly, these early synaptic aberrations are highly correlated with disease severity and cognitive decline [5–7]. Although recently developed Aβ-directed immunotherapies can somewhat slow down cognitive decline, clinical benefit is small and associated with vascular side effects including hemorrhage [8, 9]. To improve therapeutic strategies, it is therefore pivotal to better understand the physiological role of APP for synapse formation, function, and plasticity in health and disease.

APP is a type I transmembrane protein that is highly expressed across cell types in the brain, with particularly high expression during synaptogenesis. It is present at both pre- and postsynaptic sites and constitutes approximately 1% of all synaptic proteins [10–12]. The analysis of APP functions has been complicated by its highly complex proteolytic processing that leads to diverse proteolytic fragments that may each serve specific functions [13]. Within the canonical amyloidogenic pathway, APP is first cleaved by β-secretase, yielding the ectodomain APPsβ, followed by γ-secretase-mediated processing of membrane-anchored C-terminal fragments (CTFβ) to produce Aβ peptides. In the competing and physiologically predominant α-secretase pathway, cleavage by ADAM10 precludes Aβ generation and results in the secretion of APPsα. When applied exogenously to neuronal tissue, APPsα has been shown to exert neuroprotective, neurotrophic, neurogenic, and synaptic plasticity-enhancing effects (reviewed in [13, 14]). In contrast, it is well established that elevated Aβ concentrations and, in particular, oligomeric and aggregated Aβ species induce synaptic depression, spine loss, and neuronal loss [15–18]. On the other hand, in vitro studies indicated that at low concentrations, Aβ monomers may regulate synaptic vesicle recycling and transmitter release [19, 20], and were recently also found to promote synapse formation in vitro [21]. Besides being the precursor of various proteolytic fragments, there is also evidence that transmembrane APP may function as a synaptic adhesion molecule (SAM), similar to classical SAMs such as neurexin and neuroligin, by forming *trans*-directed synaptic dimers at pre- and postsynaptic sites [22–27]. When assessing APP functions using simple APP knockout (KO) mice, it is important to note that this strategy will inevitably abrogate functions mediated by transmembrane APP and, at the same time, functions mediated by the various APP proteolytic fragments. In this regard, APP-KO mice showed reduced brain and body weight [28, 29], increased susceptibility to seizures and injury [30, 31], and, most notably, age-dependent deficits in neuronal morphology, synaptic density, synaptic plasticity, and behavior [28, 32–36]. Conversely, overexpression of either wild-type APP or human APP (huAPP) variants associated with familial AD (FAD) in transgenic mice will also result in the overproduction of proteolytic fragments at non-physiological concentrations, which will not adequately mimic the physiological situation and may yield artifacts [37, 38].

Here, to unequivocally dissect the in vivo role of physiological APP processing, we generated a novel APP knockin (KI) mouse (termed APPΔS622) in which we modified the genomic mouse APP locus to delete the α- and β-secretase cleavage sites while APP transmembrane topology and the integrity of the APP C-terminus were fully preserved. Due to this deletion, APPΔS622 mice lack major proteolytic fragments such as APPsα, APPsβ, and Aβ. APPΔS622 mice therefore provide a unique model to distinguish the physiological role of endogenous APP processing from functions mediated by the transmembrane APP. In this study, we demonstrate in APPΔS622 mice an accumulation of uncleaved, full-length APP at the cell surface and at synaptic sites, along with a marked reduction in secreted APPs fragments. Electrophysiological recordings revealed a pronounced impairment in hippocampal long-term potentiation (LTP) that could be significantly rescued by recombinant APPsα (recAPPsα). At the structural level, this was accompanied by a reduction in the density of mature dendritic spines and decreased spine head size. In vitro, Aβ had also been implicated in processes of homeostatic synaptic plasticity induced by blockade of network activity using tetrodotoxin (TTX). Indeed, APPΔS622 mice lacking endogenous Aβ production displayed a loss of homeostatic synaptic plasticity that could be rescued by exogenous Aβ. Importantly, deficits in synaptic plasticity were also associated with impairments in spatial working memory. Together, our studies reveal essential roles of physiological APP processing in distinct forms of synaptic plasticity, with APPsα supporting Hebbian plasticity and Aβ enabling homeostatic synaptic plasticity, both of which are important for normal brain physiology and cognition.

## Results

### Generation of secretion-deficient APPΔS622-knockin mice lacking the α- and β-secretase cleavage sites

**Fig. 1:**
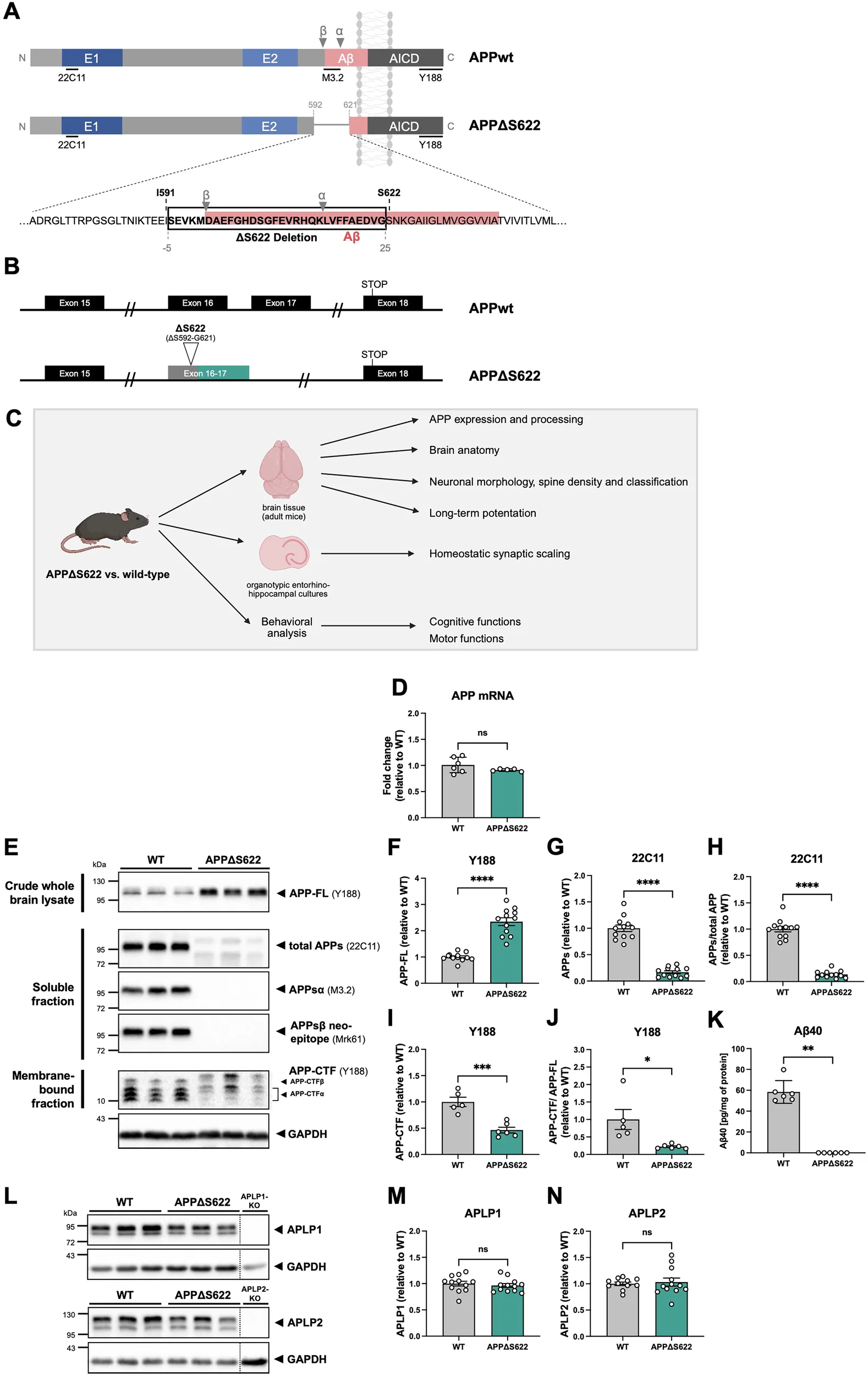
APPΔS622 mice show increased abundance of APP-FL, severely impaired APPs shedding and lack of Aβ production. **A** Schematic representation of the APPΔS622 protein variant. The deletion spans amino acids S592–G621 of APP_695_ ( − 5 to +25 relative to Aβ (red)), removing both the α- and β-secretase cleavage sites. **B** Genomic organization of the engineered APP allele. A hybrid exon 16–17 harboring the ΔS622 deletion was introduced into the endogenous *App* locus by knockin replacing exon 16 and 17, resulting in expression of the mutant APP transcript under control of the endogenous promoter. **C** Schematic representation of the experimental outline. For details on the age and sex of the animals and the cohort size, see Supplementary Fig. S2. The figure was created in BioRender. **D** qPCR analysis of APP mRNA showed no change in transcript levels between APPΔS622 and WT brain. **E** Representative Western blots. Top: whole-brain lysates of three animals per genotype analyzing full-length APP (APP-FL; C-terminal antibody Y188); middle: soluble APP species (APPs; N-terminal antibody 22C11), APPsα (M3.2) and APPsβ (Mrk61); bottom: APP C-terminal fragments (APP-CTFs; Y188). Staining with antibodies recognizing APPsα (M3.2) and APPsβ (Mrk61) revealed the absence of APPsα and APPsβ in APPΔS622 mice. See **A** for localization of antibody epitopes. GAPDH served as a loading control. **F**–**J** Quantification of APP-FL and proteolytic fragments. **F** APPΔS622 mice showed a marked accumulation of APP-FL in membrane fractions detected by Y188 (2.35-fold increase vs. WT; *n* = 12 mice per genotype). **G** Soluble APPs species were strongly reduced to 17% of WT (22C11; *n* = 12). **H** APPs/APP-FL ratio (based on 22C11) was decreased to 14% of WT (*n* = 12). **I** APP-CTFs were reduced to 46% of WT (*n* = 5–6). **J** APP-CTF/APP-FL ratio decreased to 22% of WT (*n* = 5–6). **K** Aβ40 concentration measured by MSD-ECLIA was below the detection limit in APPΔS622 brains, consistent with a complete loss of cleavage at the β-secretase site (*n* = 5). **L** Representative Western blots of APLP1 and APLP2 in whole brain lysates of three animals per genotype, with GAPDH as loading control. APLP1-KO and APLP2-KO brain samples indicate antibody specificity. Quantification of APLP1 (**M**) and APLP2 (**N**) demonstrated unchanged expression of both APP family members. Data information (**D**–**N**): Mice of both sexes with balanced numbers were used throughout (age: 3–4 months). Densitometric quantification of Western blot bands was normalized to total protein per lane determined from stain-free membrane imaging. Statistical significance was determined using an unpaired Student’s *t*-test, and data are represented as mean ± SEM. *p < 0.05, **p < 0.01, ***p < 0.001, ****p < 0.0001, and ns, not significant.

To generate mice lacking both the α- and β-secretase sites, we introduced the APPΔS622 mutation previously characterized in non-neuronal cells [23, 25] into the endogenous murine APP locus using gene targeting. To generate this novel APPΔS622-knockin (KI) variant, we replaced murine APP exons 16 and 17 with a hybrid exon 16/17 lacking APP amino acids 592-621 (APP_695_ nomenclature), corresponding to a deletion of ΔAβ (-5 to +25) (see Figs. 1A, B and S1) up to serine S622, thereby removing both the β- and α-secretase sites. Heterozygous mice were intercrossed to generate homozygous APPΔS622 mice and wild-type (WT) controls for further analysis (see Figs. 1C and S2 for experimental design). Homozygous APPΔS622 mice proved fully viable up to at least 16 months of age and revealed no obvious anatomical defects, had a normal cortical and hippocampal volume, a normally layered cortex and hippocampus, as well as no overt  signs of astro- or microgliosis (Fig. S3).

### APPΔS622 mice show severely impaired APP shedding and lack Aβ production

First, we studied the expression of the APPΔS622-KI variant at the mRNA level by qPCR. Compared to wild-type APP, APPΔS622 mRNA expression was not significantly altered (Fig. 1D). To analyze APPΔS622 protein expression and to determine whether the lack of α- and β-secretase sites increases overall APPΔS622 protein abundance, we first performed Western blot analysis using whole brain lysates. Probing blots with an antibody directed at the APP intracellular domain (Y188) revealed a pronounced accumulation of full-length APPΔS622-FL, that was increased by 2.35-fold compared to APP-WT (Fig. 1E, top and Fig. 1F; WT 1.00 ± 0.05 vs. APPΔS622 2.35 ± 0.15; unpaired Student’s *t*-test, **** *p* < 0.0001) in whole brain lysates. Next, we studied APP processing in more detail (Fig. 1E-K) and separated soluble APPs species from membrane-bound APP by ultracentrifugation (UC) of brain homogenates.

As expected from the genomic deletion, no signal was detectable in soluble fractions for APPsα using antibody M3.2, which recognizes an epitope located at the C-terminus of APPsα (Fig. 1E, middle). Likewise, APPsβ was also undetectable when probing blots with the Mrk61 antibody that selectively binds to an APPsβ-specific C-terminal neo-epitope produced upon β-secretase cleavage (Fig. 1E, middle). The lack of APPsα, APPsβ, as well as that of Aβ (see below), thus confirms correct gene targeting. Consistent with the increase in full-length APPΔS622, soluble APPs species (detected by N-terminal 22C11 antibody) were drastically reduced to only 17% compared to wild-type (WT) brain (Fig. 1G; WT 1.00 ± 0.06 vs. APPΔS622 0.17 ± 0.03; unpaired Student’s *t*-test, **** *p* < 0.0001) and the ratio of soluble APPs to total APP-FL was reduced to 14% (Fig. 1H; WT 1.00 ± 0.05 vs. APPΔS622 0.14 ± 0.02; unpaired Student’s *t*-test, **** *p* < 0.0001). Together, these data indicate a drastic decrease in shedding of soluble APPs species in APPΔS622-KI mice, due to the genomic deletion of the known α- and β-secretase sites. Further, we also analyzed C-terminal membrane-bound fragments (CTFs), which were again strongly reduced in APPΔS622 mice, although not to the same extent as APPs (Fig. 1E, bottom and Fig. 1I), likely due to the longer half-life of membrane-anchored CTFs that are more stable. The ratio of APPΔS622-CTFs to full-length APPΔS622-FL was reduced to 22% (Fig. 1J WT 1.00 ± 0.29 vs. APPΔS622 0.22 ± 0.02; unpaired Student’s *t*-test, * *p* = 0.0154). Lastly, we determined endogenous Aβ generation using a highly sensitive MSD-ECLIA. While murine Aβ1-40 was readily detectable in WT brain, amounting to 58.18 ± 4.46 pg/mg of total protein, no signal was obtained with APPΔS622 brain homogenates (Fig. 1K), thus confirming the abrogation of endogenous Aβ production.

We also studied the expression of the closely related amyloid precursor-like proteins (APLPs) to assess potential compensatory regulation in APPΔS622 mice [13, 39]. Western blot analysis of whole brain samples with knockout-validated antibodies indicated unaltered APLP1 and APLP2 expression (Fig. 1L-N).

### Increased abundance of uncleaved APP at the neuronal cell surface and at synaptic compartments of APPΔS622 mice

**Fig. 2:**
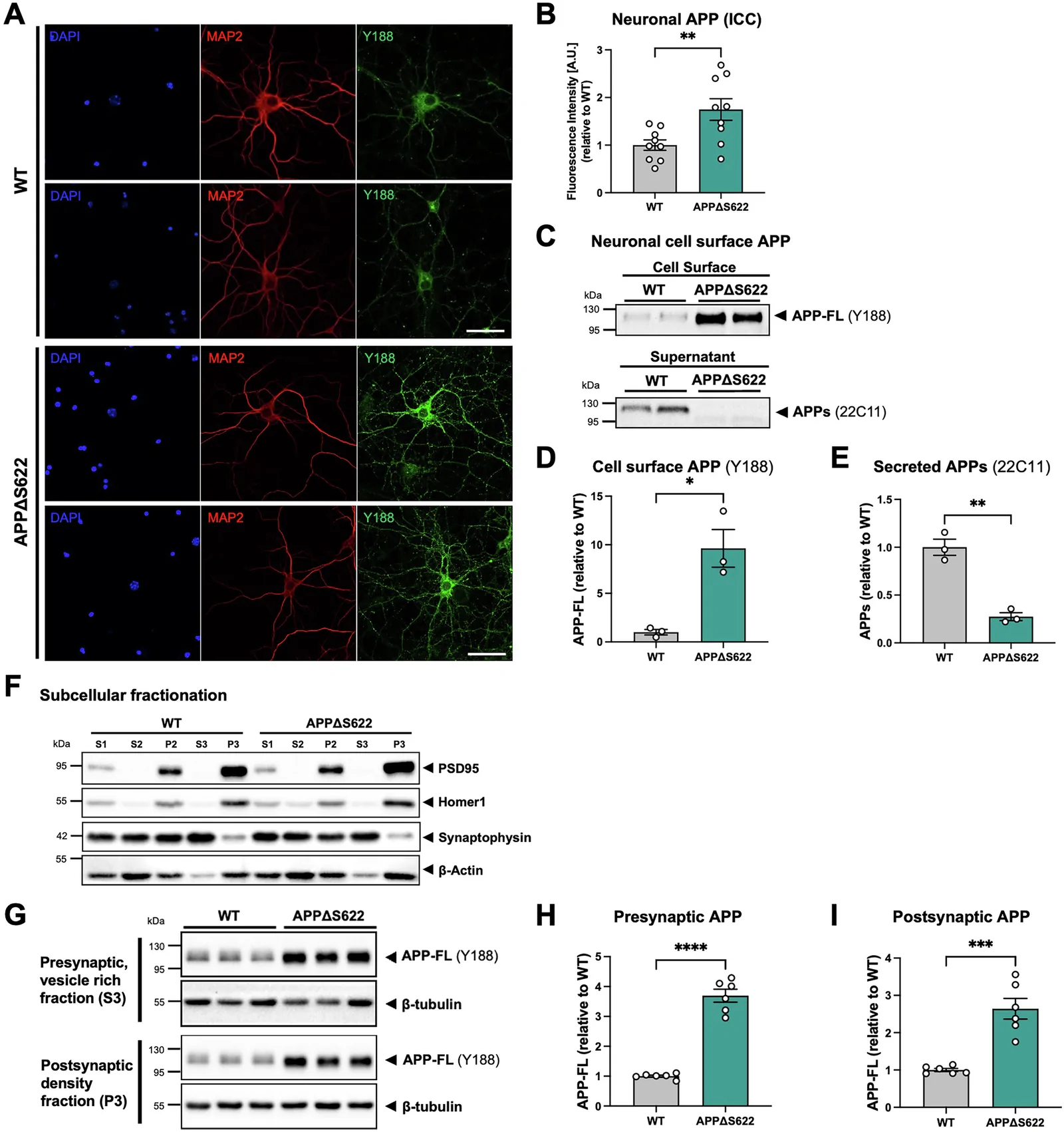
Increased abundance of uncleaved APP at the neuronal cell surface and at synaptic compartments of APPΔS622 mice. **A**, **B** Immunocytochemistry (ICC) of primary cortical neurons (DIV14) from WT and APPΔS622 mice stained for DAPI, MAP2 (red, Alexa Fluor 555), and full-length APP using the C-terminal antibody Y188 (green, Alexa Fluor 488). Confocal images were acquired on a Nikon AX microscope using identical acquisition settings across genotypes. Two representative images per genotype are shown. **B** Quantification of ICC against APP-FL. Note that APPΔS622 neurons exhibited a pronounced increase in APP staining in the soma and dendrites compared to WT neurons, indicating accumulation of full-length APP. *n* = 9 neurons from *N* = 1-2 cultures of both sexes per genotype. Scale bar = 40 µm. **C**, **D** Primary cortical neurons from WT and APPΔS622 mice were metabolically labeled with N_3_-modified tetra-acetylated N-azidoacetylmannosamine and biotinylated. Surface glycoproteins were enriched by streptavidin pulldown and subjected to immunoblotting for APP-FL. At the cell surface, APP is enriched 7.4-fold in APPΔS622 neurons compared to WT (*n* = 3 neuronal cultures of both sexes per genotype). **C**, **E** Supernatant of primary cortical neuronal cultures from WT and APPΔS622 mice was collected and concentrated to detect secreted APPs species via immunoblot. Secreted APPs was reduced to 27.5% in the medium of APPΔS622 neurons compared to WT (*n* = 3 neuronal cultures of both sexes per genotype). **F** Representative immunoblots of WT and APPΔS622 cortical brain lysate to assess the quality of synaptosomal fractionation. Subcellular fractions from synaptosome isolation: S1, post-nuclear supernatant; P2, crude synaptosomal fraction; S2, cytosolic supernatant; P3, PSD fraction; S3, presynaptic, vesicle-rich fraction. **G** Representative immunoblots of APP-FL and β-tubulin in presynaptic, synaptic vesicle–enriched fraction (S3, top) and the postsynaptic density fraction (P3, bottom) of cortical brain lysates from APPΔS622 and wild-type (WT) mice. **H**, **I** Quantification of APP-FL in synaptic subfractions S3 and P3. **H** Presynaptic APP-FL abundance was increased 3.7-fold in APPΔS622 mice (*n* = 6 animals per genotype (age: 3-4 months, both sexes)). **I** Postsynaptic APP-FL abundance was elevated 2.6-fold (*n* = 6). Data information (**E**–**I**): Densitometric quantification of Western blot bands was normalized to total protein per lane determined from stain-free membrane imaging. Data were analyzed using an unpaired Student’s *t*-test and are represented as mean ± SEM. *p < 0.05, **p < 0.01, ***p < 0.001, ****p < 0.0001.

Next, we used primary neuronal cultures to study neuronal APPΔS622 expression, surface localization, APPs secretion, and abundance after synaptosome enrichment. Consistent with Western blot analysis of whole brain lysates, immunocytochemistry using the Y188 antibody again revealed a robust increase in total APPΔS622-FL in cortical neurons (14 days in vitro (DIV14)) by about 1.75-fold (Fig. 2A, B; WT 1.00 ± 0.11 vs. APPΔS622 1.75 ± 0.23; unpaired Student’s *t*-test, ** *p* = 0.0092).

To assess the abundance of APPΔS622 at the cell surface of neurons, we employed metabolic labeling of glycosylated APP using an N_3_-modified sugar, followed by click chemistry biotinylation and streptavidin pulldown [40, 41]. Primary cortical neurons from either WT or APPΔS622 mice were cultured for 12 days in vitro (DIV12), and the azide (N_3_)-modified sugar tetra-acetylated N-azidoacetylmannosamine (ManNAz) was added to the culture medium for another 48 h. ManNAz is metabolized and incorporated as sialic acid into the glycan moieties of newly synthesized glycoproteins, including APP. Glycoproteins containing ManNAz were then covalently bound (“clicked”) to the membrane-impermeable sulfo-dibenzylcyclooctyne-biotin conjugate (DBCO), followed by streptavidin pulldown from cell lysates. Western blot analysis indicated an increased cell surface abundance of APPΔS622-FL by about 7.4-fold, while soluble APPs detected in the supernatant of neuronal cultures was again drastically decreased (Fig. 2C-E; WT 1.00 ± 0.08 vs. APPΔS622 0.27 ± 0.04; unpaired Student’s *t*-test, ** *p* = 0.0015).

Finally, we studied whether the abundance of APPΔS622-FL is not only highly increased at the neuronal cell membrane but also upregulated at the synapse. To enrich for pre- and postsynaptic proteins, we performed synaptosome fractionation (Fig. 2F). Here, we found an increase in presynaptic APPΔS622 by 3.69-fold (Fig. 2G, H; WT 1.00 ± 0.04 vs. APPΔS622 3.69 ± 0.22; unpaired Student’s *t*-test, **** *p* < 0.0001) and by 2.64-fold in postsynaptic PSD-enriched fractions (Fig. 2G, I; WT 1.00 ± 0.04 vs. APPΔS622 2.64 ± 0.28; unpaired Student’s *t*-test, *** *p* = 0.0002).

### APPΔS622 pyramidal neurons show reduced density of mature spines and smaller spine heads

**Fig. 3:**
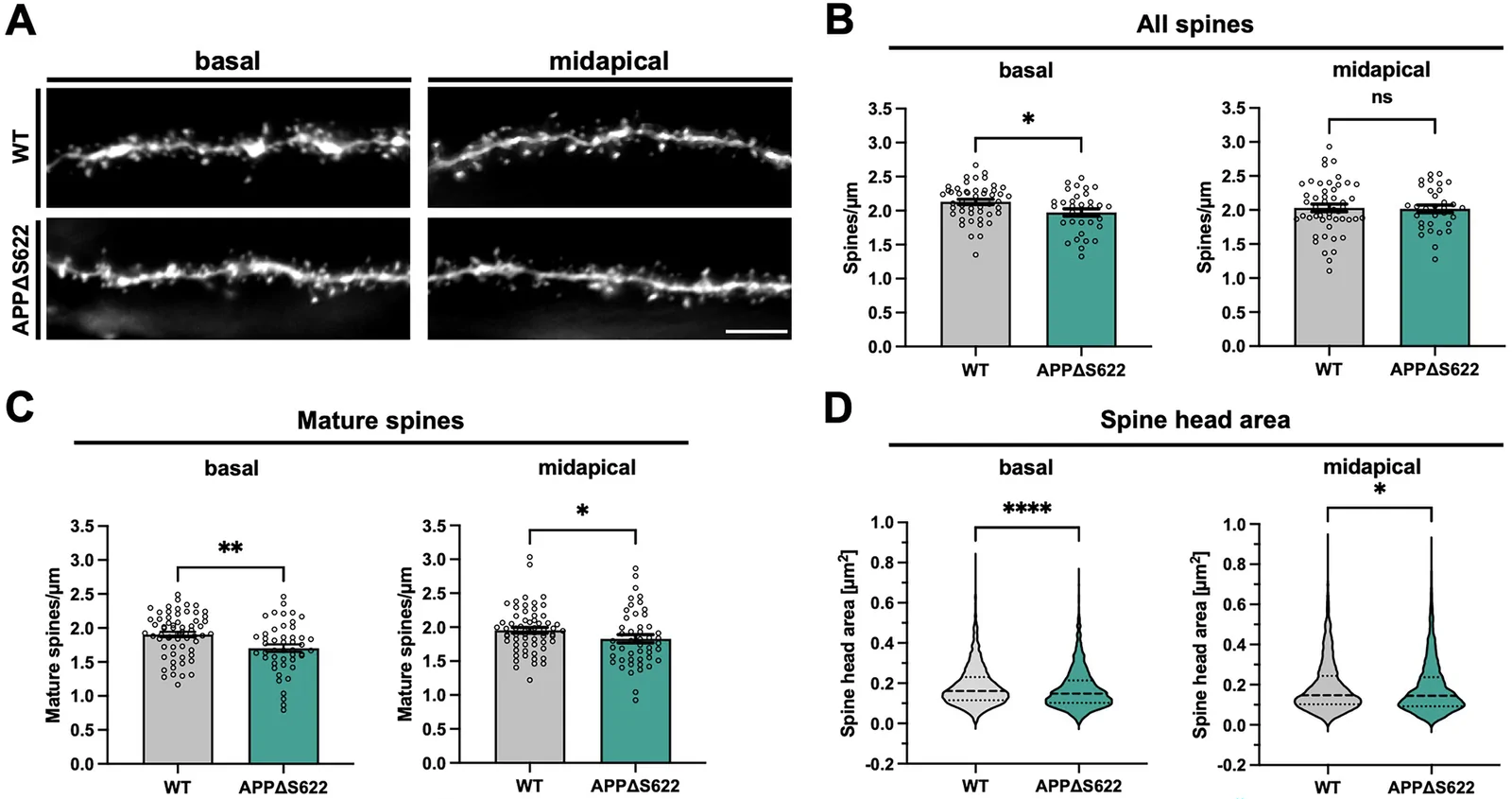
APPΔS622 pyramidal neurons show reduced density of mature spines and smaller spine heads. **A** Representative images of basal and midapical dendritic segments from CA1 hippocampal neurons of WT and APPΔS622 mice that were filled with biocytin and labelled with Alexa Fluor 594-conjugated streptavidin. Scale bar: 5 μm. **B** Overall spine density of CA1 pyramidal cells was slightly reduced in basal (WT: *n* = 48 dendritic segments, *N* = 6 animals per genotype; APPΔS622: *n* = 33, *N* = 5) and unchanged in midapical dendritic segments (WT: *n* = 49, *N* = 6; APPΔS622: *n* = 32, *N* = 5). **C** Density of mature spines is significantly reduced in CA1 pyramidal neurons of APPΔS622 mice in basal (WT: *n* = 63, *N* = 7; APPΔS622: *n* = 47, N = 6) and midapical dendritic segments (WT: *n* = 63, *N* = 7; APPΔS622: *n* = 48, *N* = 6). **D** Mature spines of basal (WT: *n* = 4460, *N* = 7; APPΔS622: *n* = 3101, *N* = 6) and midapical dendritic segments (WT: *n* = 4769, *N* = 7; APPΔS622: *n* = 3296, *N* = 6) show significantly reduced spine head area. In the violin plots, the dashed line represents the median, while the pointed lines indicate the quartiles. Data information (**B**, **C**): *n* = spine density per dendritic segment, *N* = number of animals (age: 4 months). Data passing the Shapiro-Wilk normality test were analyzed with an unpaired Student’s *t*-test with Welch’s correction, whereas non-normally distributed data were analyzed using the Mann–Whitney test. Data are represented as mean ± SEM. (**D**): *n* = mature spine heads, *N* = number of animals (age: 4 months). Mice of both sexes with balanced numbers were used throughout. Normality was assessed using the Kolmogorov–Smirnov test for larger sample sizes. Data passing normality were analyzed with an unpaired Student’s *t*-test with Welch’s correction, whereas non-normally distributed data were analyzed using the Mann–Whitney test. *p < 0.05, **p < 0.01, ****p < 0.0001, and ns, not significant.

Next, we studied the morphology of hippocampal neurons. We focused on CA1 neurons because this region is highly vulnerable in AD, is one of the best studied brain regions with regard to synaptic plasticity, and previous analyses indicated LTP defects at CA3/CA1 synapses, altered dendritic architecture, and altered spine density in APP knockout mice [28, 32, 34, 35, 42]. For Sholl analysis, biocytin-filled CA1 neurons of young adult mice (age: 3-4 months) were imaged and their dendritic tree was reconstructed (Fig. S4). We plotted the number of intersections (measured within circles centered on the soma, see Fig. S4A) against the distance from the soma and analyzed basal and apical dendrites separately. When comparing CA1 neurons of both genotypes (APPΔS622 and WT littermate (LM)), we detected no significant difference in the complexity of either basal or apical dendrites (Fig. S4B, G), in the number of primary basal dendrites (Fig. S4C), in total dendritic length (Fig. S4D, H), and in the total number of intersections with Sholl spheres or nodes (Fig. S4E, F and I, J). This indicates that increased abundance of APP at the cell surface and the lack of APPsα/Aβ do not affect overall neuronal morphology. We then went on to assess spine density as a correlate of excitatory synapses. Spine counts were performed separately in midapical portions of apical dendrites (a region densely innervated by CA3 cells) and in basal dendrites, using either biocytin-filled neurons (Fig. 3), or Golgi-staining (see Fig. 6). In young adult mice (age: 3-4 months) a subtle reduction in spine density was observed in basal dendrites of biocytin-filled neurons (Fig. 3B; WT 2.13 ± 0.04 spines/µm vs. APPΔS622 1.97 ± 0.05 spines/µm; unpaired Student’s *t*-test with Welch’s correction, * *p* = 0.0190); however, this effect was not evident in midapical dendritic segments, despite the robust overexpression of APPΔS622 at the cell surface and in synaptic compartments. This indicates that APPΔS622 overexpression and presumably increased synaptic adhesion does not lead to an increase, but rather a reduction in hippocampal spine density in vivo. On the other hand, APPΔS622 mice lack APPsα, which has been functionally linked to LTP, a process of Hebbian synaptic plasticity known to induce the growth of potentiated spine heads and stabilization of mature spines. We therefore wondered whether mature spines might be specifically affected in APPΔS622 mice. Indeed, when we focused our analysis on the density of mature spines—classified as spines with a discernible spine head—we found a significant decrease in mature spines in both basal (WT 1.90 ± 0.04 spines/µm vs. APPΔS622 1.70 ± 0.05 spines/µm; unpaired Student’s *t*-test with Welch’s correction, ** *p* = 0.0038) and apical dendrites (WT 1.95 ± 0.04 spines/µm vs. APPΔS622 1.83 ± 0.06 spines/µm; Mann-Whitney test, * *p* = 0.0394) of APPΔS622 CA1 neurons (Fig. 3C). Moreover, the spine head area of mature spines was significantly smaller in CA1 pyramidal neurons of APPΔS622 mice (Fig. 3D), as compared to wild-type controls (Fig. 3D). Together, this indicates that the APPΔS622 mutation impairs the size and density of mature spines.

### Lack of APP processing impairs long-term potentiation and can be rescued by recAPPsα application

**Fig. 4:**
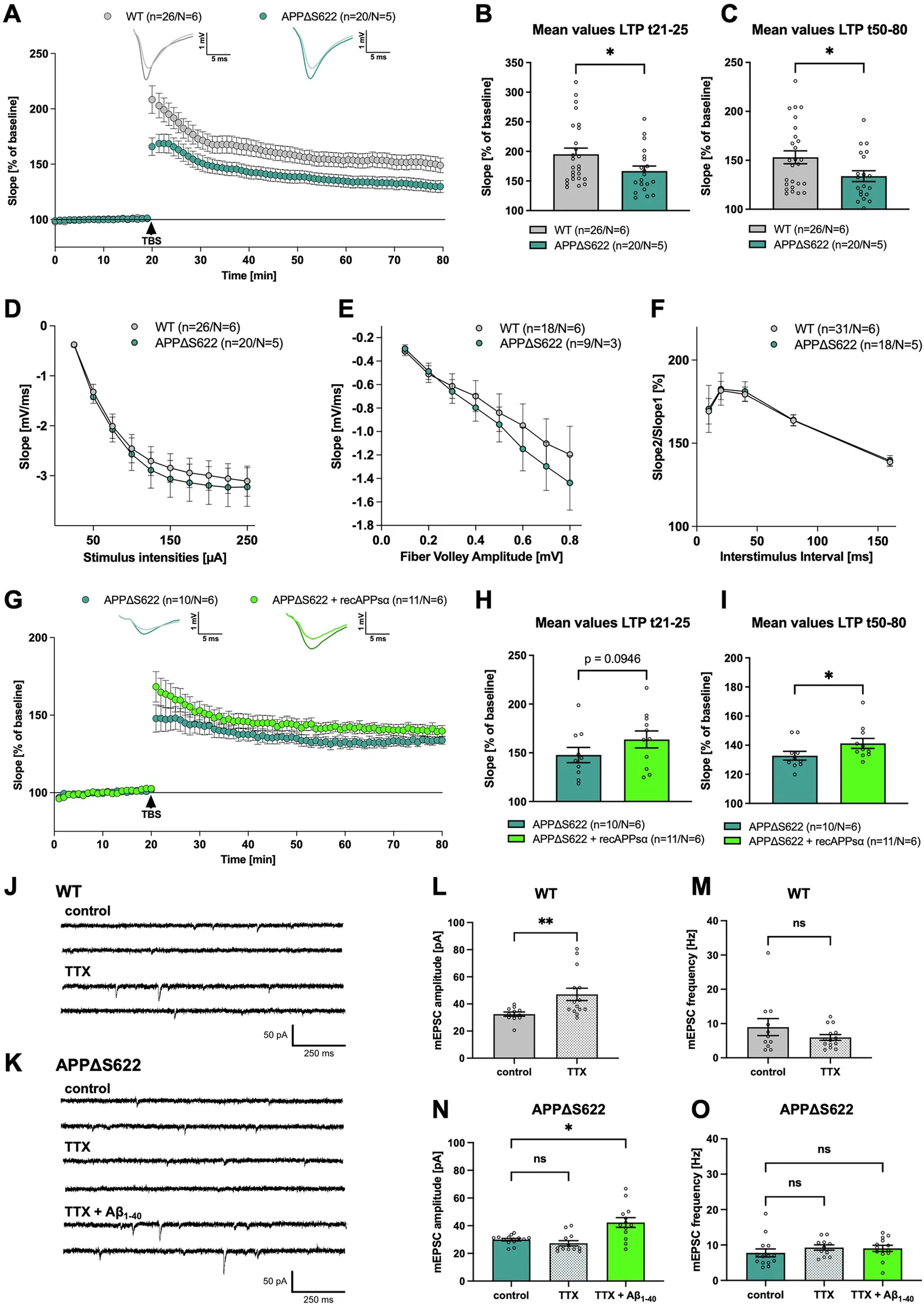
APPΔS622 mice exhibit impaired hippocampal LTP that is rescued by acute recombinant APPsα application and deficits in homeostatic synaptic plasticity that are rescued by Aβ. **A**–**C** Long-term potentiation (LTP) was induced at Schaffer collateral CA3–CA1 synapses following a 20 min baseline recording using theta-burst stimulation (TBS; arrowhead). **B** During LTP induction, APPΔS622 slices displayed significantly reduced potentiation compared to WT. (**C**) Synaptic efficacy remained impaired in the maintenance phase, with reduced potentiation during the final 30 min of recording. **D**, **E** Basal synaptic transmission was unaltered in APPΔS622 mice. **D** The correlation of given stimulus intensities to fEPSP slope was not significantly different between genotypes. **E** The correlation of fEPSP slope to defined fiber volley amplitudes revealed unaltered IO strength. **F** Paired-pulse facilitation revealed normal short-term plasticity. **G**–**I** Acute application of 10 nM recombinant APPsα rescued the LTP deficit in APPΔS622 slices. **H** At LTP induction, a trend toward improved potentiation was observed. **I** During the maintenance phase of LTP, synaptic efficacy was significantly enhanced in APPΔS622 slices treated with recombinant APPsα. **J**–**O** Analysis of homeostatic synaptic plasticity in organotypic entorhonal-hippocampal cultures of APPΔS622 mice: **J**, **L**, **M** Sample traces and group data of AMPAR-mediated mEPSCs recorded from CA1 hippocampal neurons in vehicle-treated (control) and TTX-treated WT cultures (control, *n* = 11 cells from *N* = 4 cultures; TTX, *n* = 14 cells from *N* = 5 cultures). TTX treatment led to an increase in mEPSC amplitude in CA1 hippocampal neurons from WT tissue cultures. **K**, **N**, **O** Sample traces and group data of AMPAR-mediated mEPSCs recorded from CA1 hippocampal neurons in vehicle-treated (control), TTX-treated, and TTX + Aβ_1-40_-treated APPΔS622 cultures (control, *n* = 14 cells from *N* = 5 cultures; TTX, *n* = 12 cells from *N* = 4 cultures; TTX + Aβ_1-40_, *n* = 13 cells from *N* = 5 cultures). TTX treatment did not lead to an increase in mEPSC amplitude in CA1 hippocampal neurons from APPΔS622 cultures. In the presence of Aβ_1-40_ (1.5 µM), homeostatic synaptic plasticity was rescued in APPΔS622 neurons. Data information: (**A**–**I**): *n* = number of slices, *N* = number of animals (age: 4–5 months). Slices from mice of both sexes with balanced numbers were used throughout. Normality was assessed using the Kolmogorov–Smirnov test. Data passing normality were analyzed with an unpaired Student’s *t*-test, whereas non-normally distributed data were analyzed using the Mann–Whitney test. **J**–**O**: Cultures from mice of both sexes were used throughout. Data were analyzed using the Mann–Whitney test (**L**, **M**) and, for multiple group comparisons, using the Kruskal–Wallis test with Dunn’s *post hoc* test. Data are represented as mean ± SEM. *p < 0.05, **p < 0.01, and ns, not significant.

To assess whether deficits in the density of mature dendritic spines and reduced spine head area are associated with alterations in synaptic and network activity of the hippocampus, we first analyzed activity-dependent synaptic plasticity. Previous studies had indicated an important role of APPsα for LTP, as exogenous application of recAPPsα or AAV-mediated APPsα overexpression efficiently rescued hippocampal LTP deficits in forebrain-specific cDKO mice lacking APP and the close homologue APLP2 [43, 44], and also in AD model mice [45–48]. However, whether endogenously produced APPsα is essential for normal synaptic plasticity has remained unknown. We therefore performed extracellular field recordings and examined LTP in hippocampal slices of young adult APPΔS622 mice (age: 4-5 months) versus wild-type littermate controls (Fig. 4A-C). After 20 min of stable baseline recording, LTP was induced at the CA3/CA1 (Schaffer collateral) pathway by application of theta-burst stimulation (TBS, arrowhead Fig. 4A). Strikingly, LTP was significantly reduced in APPΔS622 mice, both immediately after LTP induction (see Fig. 4B; t20-t25: LM control 195.2 ± 10.30% vs. APPΔS622 166.8 ± 8.40%; Mann-Whitney test, * *p* = 0.0492) and also during LTP maintenance (potentiation in LM mice at t50-80, 30 min after TBS: 153 ± 6.59% vs APPΔS622 133.8 ± 5.49% Mann-Whitney test, * *p* = 0.0395). To investigate whether the observed LTP defect might be due to disturbed basal synaptic transmission, we examined pre- and postsynaptic functionality. Comparing the field excitatory postsynaptic potential (fEPSP) size at defined stimulus intensities revealed no significant difference between the two genotypes (Fig. 4D). Further, we failed to detect differences in input-output strength at given fiber volley amplitudes (Fig. 4E). Likewise, presynaptic functionality also appeared unaffected when using the paired-pulse facilitation (PPF) paradigm (Fig. 4F). In a separate set of experiments, we asked whether LTP deficits associated with the absence of endogenous APP fragments, including APPsα, could be rescued by exogenous APPsα application. Indeed, application of 10 nM recAPPsα, a concentration we and others had successfully used in previous studies (see [43, 44, 49]), significantly enhanced LTP in APPΔS622 mice (Fig. 4I; t50-80: untreated APPΔS622 132.8 ± 3.00% vs APPΔS622 ± recAPPsα 141.2 ± 3.46%; Mann-Whitney test, * *p* = 0.0215). Together, these data indicate normal basal synaptic physiology of APPΔS622 mice and a sufficiency of APPsα to rescue LTP, which suggests that endogenously produced APPsα is required for normal LTP.

### Aβ restores homeostatic synaptic plasticity in APPΔS622 slice cultures

Whereas Hebbian synaptic plasticity can be induced by brief patterns of activity, non-Hebbian homeostatic synaptic plasticity is triggered by prolonged changes in neural activity. For example, chronic silencing with tetrodotoxin (TTX) induces a compensatory upscaling of excitatory synaptic strength [50, 51]. We previously showed that amyloidogenic APP processing, and specifically Aβ rather than APPsα, is required for TTX-induced homeostatic synaptic plasticity in dentate gyrus granule cells of organotypic entorhino-hippocampal cultures (OHCs) [52]. Because APPΔS622 mice lack endogenous Aβ production, we examined homeostatic synaptic plasticity in entorhino-hippocampal cultures prepared from APPΔS622 and age/time-matched wild-type littermate controls (Fig. 4J-O). Cultures were maintained for more than 18 DIV and treated with TTX (2 µM; 2 d) to suppress network activity. AMPAR-mediated miniature excitatory postsynaptic currents (mEPSCs) were subsequently recorded from individual CA1 pyramidal cells to assess compensatory changes in excitatory synaptic strength. Consistent with our previous results in dentate gyrus granule cells [52], TTX treatment induced a robust increase in mean mEPSC amplitude in wild-type CA1 neurons (Fig. 4J, L; vehicle-treated: 32.50 ± 1.56 pA vs. TTX-treated: 47.02 ± 4.53 pA; Mann–Whitney test, ** *p* = 0.0062), indicating a homeostatic upscaling of excitatory synaptic strength. In contrast, no significant homeostatic scaling response was observed in APPΔS622 cultures (Fig. 4K, N; vehicle-treated APPΔS622 29.92 ± 0.86 pA vs. TTX 27.28 ± 1.92 pA, ns, *p* = 0.6453). Mean mEPSC frequency remained unchanged across treatments and genotypes (Fig. 4M, O).

To determine whether exogenous Aβ could restore the impaired homeostatic response, we performed rescue experiments using 1.5 µM of synthetic Aβ_1-40_, a concentration previously shown to restore TTX-induced homeostatic synaptic scaling in APP-KO organotypic slice cultures [52]. In that study, rescue was also observed with Aβ_1-42_ over a broad concentration range, including 0.5 µM, 200 nM, and 200 pM, indicating that the effect was not restricted to a single peptide concentration [52]. Indeed, co-application of TTX and Aβ_1-40_ restored the increase in mEPSC amplitude in APPΔS622 CA1 neurons (Fig. 4N; vehicle treated APPΔS622 29.92 ± 0.86 pA vs. TTX 27.28 ± 1.92 pA, ns, *p* = 0.6453 and vehicle treated APPΔS622: 29.92 ± 0.86 pA vs. TTX + Aβ_1-40_ 42.25 ± 3.50 pA, Kruskal–Wallis test followed by Dunn’s *post hoc* test, * *p* = 0.0444). The magnitude of the response was comparable to that observed in TTX-treated WT cultures.

Together, these findings show that impaired APP processing in APPΔS622 preparations is associated with a loss of TTX-induced homeostatic synaptic scaling and that this response can be restored by exogenous Aβ. In combination with our previous mechanistic work [52], the data support differential roles of APP-derived fragments in complementary forms of synaptic plasticity, with APPsα supporting hippocampal LTP and Aβ contributing to homeostatic synaptic scaling.

### APPΔS622 knockin mice show impairments in cognition

**Fig. 5:**
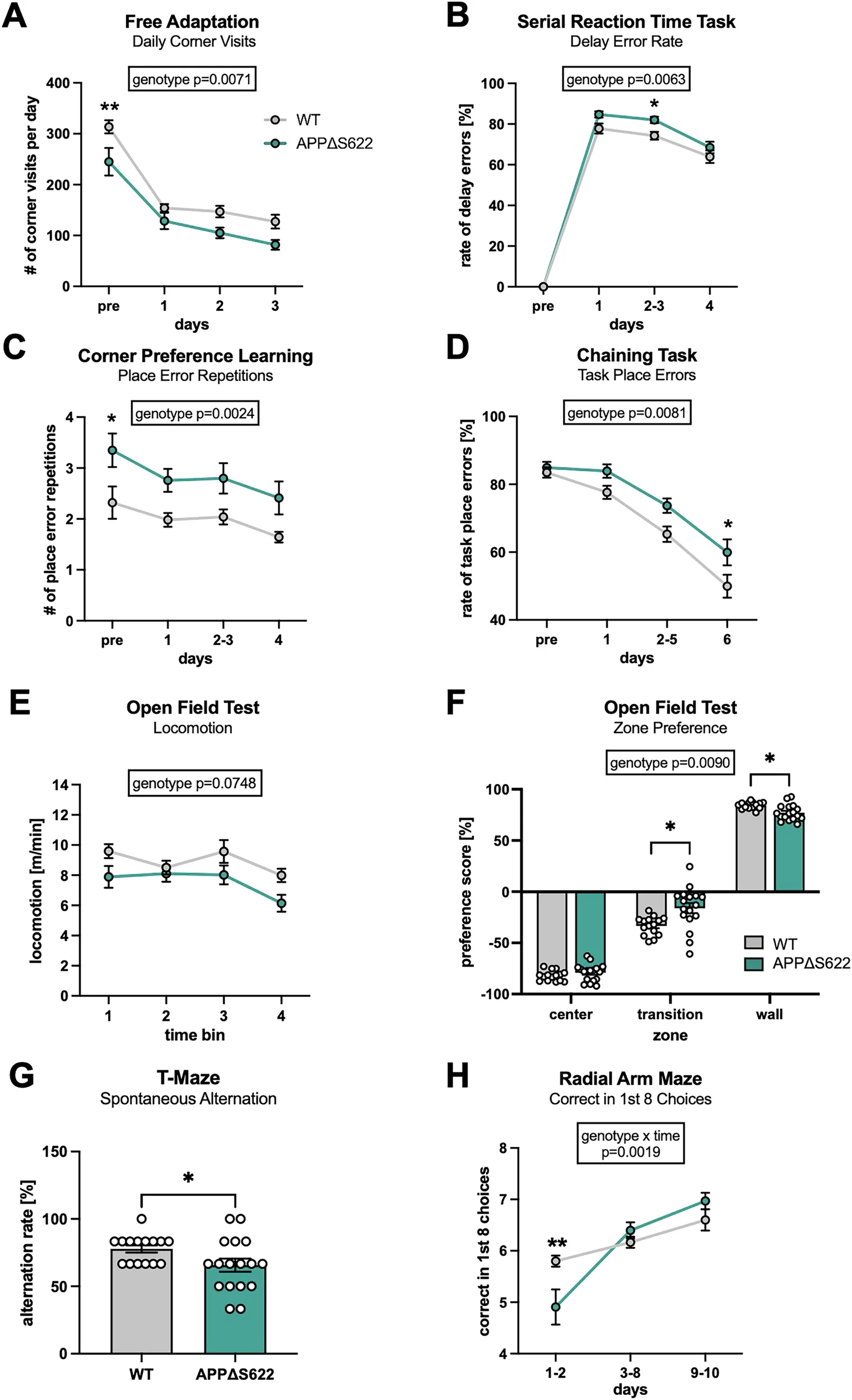
APPΔS622 knockin mice show impairments in cognition. **A**–**E** Behavioral testing in the IntelliCage (IC). **A** Daily numbers of corner visits during free adaptation as a function of time (bins: pre = first 24 h exploration of IC, 1-3 = days of habituation). APPΔS622 mutants made fewer visits than controls throughout. **B** Serial reaction time task. Rate of delay errors during serial reaction time testing as a function of time (bins: pre = last 24 h of baseline, 1-3 = training days 1, 2-3, 4). Overall, the rate of delay errors decreased robustly across days of training but remained at a high level. Delay error rate was higher in APPΔS622 mutants than in controls. **C** Corner preference learning. Place error repetitions (average number of nose pokes during responses in incorrect corners) during the corner preference task plotted as a function of time (bins: pre = last 24 h of drinking session adaptation, 1-3 = acquisition days 1, 2-3, 4). Repetitions were more numerous in APPΔS622 mutants compared to controls. **D** Chaining task. Rate of task place errors (responses in an incorrect corner during drinking sessions) during the chaining task plotted as a function of time (bins: pre = last 24 h of drinking session adaptation, 1-3 = acquisition days 1, 2-5, 6). APPΔS622 mutants made more place errors throughout testing. **E** Locomotor activity in the open field, assessed as distance moved (normalized to 1 min observation time) during two consecutive time bins of 5 min on two successive days. There was no evidence for a significant genotype effect on locomotor activity. **F** Preference scores for the center field, transition zone and wall zone in the large open field: 0 = dwell time as expected by chance, 100 = all time spent in zone, −100 = complete avoidance, average across all time bins. Aged APPΔS622 mice spent more time in the transition zone at the expense of the wall zone. **G** Spontaneous alternation rates during six runs in a T-maze. Overall, controls showed robust alternation, whereas there was a significant reduction in aged APPΔS622 mice. **H** Correct responses among the first 8 choices in the 8-arm radial-maze. The mice did one trial per day. Overall, the mice showed robust learning. Aged APPΔS622 mice earned lower scores during the first two days of training compared to age-matched WT littermates. Data were analyzed using two-way ANOVA with Bonferroni’s multiple comparisons *post hoc* test. Data information (**A**–**D**): *n* = 13-16 female mice/genotype (age: 7-9 months). **E**–**H**: *n* = 15-17 mice of both sexes/genotype (age: 13-14 months). Data are represented as mean ± SEM. *p < 0.05, **p < 0.01.

To assess whether deficits in structural and functional synaptic plasticity were also reflected in behavioral impairments, female mice were tested in the IntelliCage (IC), which permits sensitive automated testing for spontaneous behavior and various spatial learning paradigms (Fig. 5A-E and see Supplementary Fig. S5 for a schematic representation of tests). Individual mice can be tracked and are tested in a social context without exposure to novelty and without handling by the experimenter. We restricted our analysis to female mice as these can be readily housed in larger groups. During the first week of testing (free adaptation), water was freely available in all four learning corners of the IC (Fig. 5A). All mice showed robust exploration of the cage in the first 24 h followed by habituation during the next three days. During free adaptation, APPΔS622 mutants showed a very mild reduction in overall activity and made significantly fewer corner visits than controls (Fig. 5A; geno *F*(1,29) = 8.395, *p* = 0.0071; time *F*(3,87) = 90.28, *p* < 0.0001; geno x time *F*(3,87) = 0.6411, ns). Subsequently, access to water was blocked by doors that could be opened with a nose poke for 5 s to allow drinking. After nose poke adaptation, mice underwent serial reaction time testing to study impulse control. In this paradigm, the first nose poke during a corner visit initiates a delay period after which LEDs are switched on, and the door opens for drinking after a second nose poke. Premature nose pokes during the delay period are considered delay errors. For four days, the delay period varied randomly between 0.5, 1.5, and 2.5 s, and premature nose pokes had no consequence with regard to the availability of drinking water. As the mice learned, delay error rates decreased across days in both groups. APPΔS622 mutants made more delay errors as compared to controls (Fig. 5B; geno *F*(1,27) = 8.794, *p* = 0.0063; time *F*(3,81) = 826.3, *p* < 0.0001; geno x time *F*(3,81) = 1.688, ns), indicating impaired inhibition of inappropriate nose pokes. After completion of the serial reaction time task and three days of subsequent nose poke adaptation, access to water was restricted to only four daily drinking sessions (at 2-3 h, 11–12 h, 14-15 h, and 23-24 h, Supplementary Fig. S5), in which doors opened in response to nose pokes. In order to study spatial learning, mice underwent a series of hippocampus-dependent test paradigms of increasing difficulty [53, 54]. First, each mouse was assigned a single corner in the IC where it had to obtain its water by a nose poke during drinking sessions (corner preference task, see Fig. 5C and Supplementary Fig. S5). Although both groups of mice rapidly learned this simple task and made similar numbers of responses in incorrect corners, APPΔS622 mice made more repeated nose pokes than controls after not obtaining water in incorrect corners (geno *F*(1,25) = 11.40, *p* = 0.0024; time *F*(3,75) = 6.595, *p* = 0.0005; geno x time *F*(3,75) = 0.3687, ns), again indicating impaired ability to withhold inappropriate nose pokes.

Finally, mice were tested in a more demanding chaining task in which access to water changes to the next clockwise (or anticlockwise) corner after each rewarded visit. APPΔS622 mice were impaired in learning this rule and had an increased rate of place errors throughout the testing period (Fig. 5E; geno *F*(1,23) = 8.403, *p* = 0.0081; time *F*(3,69) = 85.82, *p* < 0.0001; geno x time *F*(3,69) = 1.334, ns), indicating a deficit in spatial working memory which is challenged by this task.

### APPΔS622 knockin mice show aging-related deficits in spine density associated with impairments in cognition

Finally, as IC testing was performed only in female mice, which are more tolerant to group housing, we also tested mice of both sexes that had now reached an age of 12-14 months in conventional behavioral tests. First, open field testing was performed to assess locomotion and exploratory behavior` as a baseline for subsequent tests. Overall locomotion was not significantly different between genotypes, and both groups showed habituation over time (Fig. 5F). More detailed analysis indicated that APPΔS622 mice showed a slightly reduced preference to spend time along the wall and a reduced preference for the transition zone between the center and wall of the open field, possibly indicating less cautious behavior (Fig. 5G). Normal locomotion in the OF was also consistent with unaltered performance in rotarod testing and normal grip strength, thus excluding impairments in muscle strength and motor coordination (Fig. S6). When tested in the T-maze, which assesses working memory, APPΔS622 mice were significantly impaired (Fig. 5H; WT 77.78 ± 2.66% vs. APPΔS622 65.69 ± 4.84%; Mann-Whitney test, * *p* = 0.0411). Also in the fully baited radial arm maze, APPΔS622 mice revealed impairments with fewer correct arm choices when collecting baits (Fig. 5H; time *F*(2,58) = 30.86, *p* < 0.0001, geno x time *F*(2,58) = 7.008, *p* = 0.0019), particularly on the first day of testing.

**Fig. 6:**
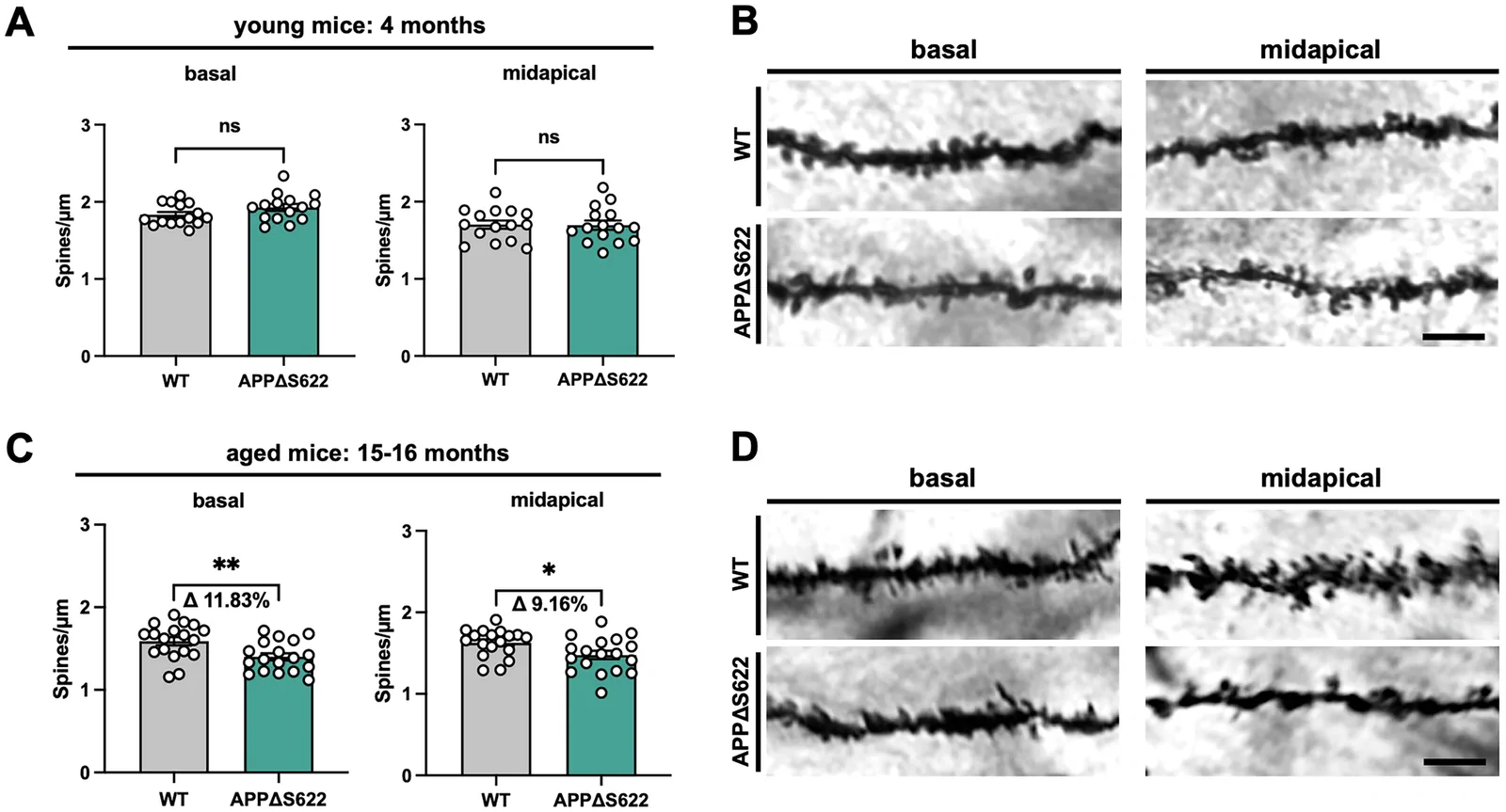
Loss of APP processing leads to reduced dendritic spine density in aged APPΔS622 mice. **A** Spine density of CA1 pyramidal cells is not altered in basal and midapical dendritic segments of young WT and APPΔS622 mice (age: 4 months, mixed sex, *n* = 15 dendritic segments, *N* = 5 animals per genotype). **B** Representative images of Golgi-stained basal and midapical dendritic segments from CA1 hippocampal neurons of young WT and APPΔS622 mice. **C** In aged APPΔS622 mice, overall spine density of CA1 pyramidal cells is reduced in basal and midapical dendritic segments (age: 15-16 months, mixed sex, *n* = 18, *N* = 6). **D** Representative images of Golgi-stained basal and midapical dendritic segments from CA1 hippocampal neurons of aged WT and APPΔS622 mice. Data information: *n* = mean spine density per neuron, *N* = number of animals. Data were analyzed using an unpaired Student’s *t*-test and are represented as mean ± SEM. *p < 0.05, **p < 0.01, and ns, not significant. Scale bar: 5 μm.

Previous data from APP-KO mice [33, 35] had indicated age-dependent impairments in spine density in constitutive APP-KO mice. We therefore used Golgi staining to assess spine density in aged mice that had undergone behavioral testing (age: 15-16 months old). Compared to WT controls, aged APPΔS622 mice revealed impairments in spine density (Fig. 6C, D), while young mice (age: 4 months old) showed no significant differences (Fig. 6A, B), consistent with previous experiments using biocytin/streptavidin staining (Fig. 3B). Together, this corroborates aging-related deficits in spine density that were associated with reduced working memory and spatial learning of APPΔS622 mice irrespective of sex.

## Discussion

Numerous APP transgenic mouse models, with diverse familial AD mutations, have been developed to recapitulate Aβ plaque pathology. In contrast, the question of whether endogenous APP processing serves an essential role in normal brain function, synaptic plasticity, and behavior has remained unresolved. This has been due to the lack of a suitable mouse model allowing for disentangling APP-FL expression from the concomitant generation of APP fragments.

Here, we generated a novel APP knockin mouse that shows largely abrogated APPs secretion, completely lacks APPsα and APPsβ, and is also deficient in the production of Aβ peptides. To this end, we deleted at the genomic level APP residues S592-G621, encompassing the endogenous α- and β-secretase sites. To specifically study the role of APP processing and secretion, it was important that the novel APPΔS622 variant preserves the transmembrane topology of APP, including the large extracellular domain that mediates transsynaptic dimerization and adhesion at central and peripheral synapses via interaction with APP, the related APLPs [22, 24, 25, 27, 39, 55], or Neurexin [23]. In addition, the APP N-terminal ectodomain (in particular the E1 domain) has been shown to interact with numerous other synaptic proteins including the closely related APLPs, members of the LDL receptor family, NMDA-R, and GABA_B_-R (reviewed in [13]). The engineered deletion also preserves the integrity of the C-terminus that controls cell surface localization, subcellular trafficking, and interactions with the presynaptic release machinery [56, 57] as well as multiple intracellular adapter molecules (e.g., via the YENTPY motif) to mediate signaling functions [13, 58, 59].

Consequently, these APPΔS622 mice allowed us to assess the physiological role of major endogenous APP processing pathways and cleavage products.

Our findings indicate that suppression of APP shedding and Aβ production does not impair synaptogenesis, as evidenced by normal overall spine density in dendrites of CA1 neurons of young APPΔS622 mice, implying that neither the secreted APPsα fragment nor Aβ are essential for the formation of excitatory synapses. On the other hand, we also did not observe an increase in spine density, as might have been expected from the increased cell surface expression of APP and the increased abundance at pre- and postsynaptic specializations that may enhance trans-synaptic adhesion and thus synapse formation. This contrasts with findings from in vitro studies that found increased spine density upon APP overexpression in cultured mouse hippocampal neurons [33]. Likewise, in a synapse formation assay in which HEK cells are co-cultured with primary neurons, expression of the secretion-deficient APPΔS622 mutant induced an increase in the number of presynaptic or postsynaptic specializations compared to APP-WT in contacting axons, or dendrites, respectively [25,27]. In addition to membrane-bound APP, non-aggregated Aβ species have also been shown to be synaptogenic in vitro when added to human iPSC-derived neurons [21]. Together, this highlights the limitations of in vitro systems and suggests that APP adhesive functions, or those mediated by monomeric Aβ, may be more relevant during early stages of neuronal differentiation and synaptogenesis. Our data also imply that synapse density is under tight control in vivo to maintain neuronal circuitry. Consistently, we found no detectable difference in total neurite length and dendritic complexity of CA1 cells from APPΔS622 mice, as assessed by Sholl analysis.

The notion that transgenic overexpression must be viewed with caution and does not adequately reflect the physiological situation is also evident from the contrasting phenotype of mice overexpressing human APP mutated at the α-secretase site (APP-R609D, K612E). These mice exhibited reduced sensitivity to NMDA, neurodegeneration, increased aggression, spontaneous seizures, and premature death in a transgene dose-dependent manner [60, 61].

A key finding of this study was that APPΔS622 mice exhibit pronounced impairments of both Hebbian (LTP) and homeostatic forms of synaptic plasticity. Lack of endogenous APP processing in APPΔS622 mice kept baseline synaptic transmission unaffected but led to impaired LTP at Schaffer collateral-CA1 synapses. Given that presynaptic functionality (assessed by the paired-pulse paradigm) was normal, LTP deficits are likely due to postsynaptic alterations. At the cellular level, LTP has been shown to strengthen synaptic weight of specific inputs via the rapid insertion and clustering of AMPA receptors (AMPARs) into the postsynaptic membrane, which in turn leads to spine enlargement and stabilization [62, 63]. In line with this, pyramidal CA1 neurons of young APPΔS622 mice revealed, despite normal overall spine density, a significantly reduced density of mature spines with reduced spine head size, suggesting that APP processing is important for structural synaptic plasticity. Interestingly, the impairment in LTP could be partially rescued by addition of recombinant recAPPsα, thus corroborating a major role of physiological APP processing and endogenous APPsα secretion for LTP. This aligns well with previous studies in wild-type rat hippocampal slices and neurons [64, 65], indicating that recAPPsα facilitates LTP by stimulating the synthesis and trafficking of GluA1-containing AMPARs, known to be important for spine enlargement [66], to extrasynaptic sites from which they can be recruited during LTP.

A limitation of our study is that we cannot formally exclude that increased abundance of full-length APPΔS622 or developmental adaptations may contribute to the phenotypic abnormalities of APPΔS622 mice. In this regard, it is noteworthy that complementary independent loss-of-function studies have previously been performed in wild-type animals [20, 49] that further support a crucial role of APPsα. Intrahippocampal infusion of antibodies directed against APPsα as well as infusion of the α-secretase inhibitor TAPI impaired LTP and spatial memory in wild-type rats [49]. The impairment of LTP could be rescued by exogenous recAPPsα, but not recAPPsβ, highlighting a central role of non-amyloidogenic APP processing for Hebbian synaptic plasticity [49]. This notion is further supported by the phenotype of APPsα knockin mice in which we inserted a stop codon behind the α-secretase site, which leads to APPsα secretion in the absence of APP-FL and Aβ expression [28]. APPsα knockin prevented the occurrence of LTP deficits that were observed in aged APP-KO mice [28], and also proved sufficient for normal spine density [28, 42, 57].

Processes of synaptic plasticity include associative, activity-dependent alterations in synaptic strength that are thought to be cellular correlates of learning and memory processes and also homeostatic synaptic plasticity that stabilizes network activity. The latter refers to a global up- and downregulation of AMPAR responses to preserve the proportional strength of individual synapses [50, 67, 68]. Interestingly, dysregulated homeostatic synaptic plasticity has also been implicated in AD and other neurodegenerative disorders [69–72]. Here, using secretion-deficient APPΔS622 mice, we demonstrate that APP processing is also essential for homeostatic synaptic scaling at hippocampal CA1 synapses. In this regard, it is important that deficits in homeostatic scaling are not due to major structural or functional alterations, as mean overall spine density and mean mEPSC amplitudes and frequencies of APPΔS622 CA1 neurons were comparable to WT littermates. Importantly, our results clearly indicate that different proteolytic fragments are required for each form of synaptic plasticity. While LTP impairments in APPΔS622 mice could be rescued by APPsα, homeostatic scaling was restored by exogenous Aβ. This finding confirms and extends our previous work in dentate gyrus granule cells of APP-KO organotypic entorhino-hippocampal cultures [52]. In this study, Aβ restored TTX-induced homeostatic synaptic scaling, whereas recombinant APPsα (10 nM), AAV-mediated neuronal APPsα expression, and constitutive APPsα knockin tissue lacking endogenous APP-FL and Aβ all failed to rescue the scaling response. Conversely, antibody-mediated depletion of endogenous APPsα did not impair homeostatic synaptic scaling in wild-type cultures [52]. Together, these complementary gain- and loss-of-function experiments indicate that APPsα is neither sufficient nor required for TTX-induced homeostatic synaptic scaling under these experimental conditions and support a specific role of Aβ in this form of plasticity. Consistent with our previous observations, the loss of homeostatic scaling in APPΔS622 mice supports a physiological role of Aβ within the signaling machinery that mediates homeostatic synaptic plasticity. Aβ may not act as the primary instructive signal but instead may function as a permissive or modulatory component of a broader homeostatic feedback pathway. Together with our previous mechanistic work, the present findings provide converging evidence that distinct APP processing products differentially regulate complementary forms of synaptic plasticity required for network stability and normal brain function.

Synaptic-weight-based Hebbian plasticity and homeostatic synaptic scaling are key mechanisms that enable memory while stabilizing neuronal networks. Indeed, impairments in synaptic plasticity of APPΔS622 mice also translated into significant functional impairments in three behavioral tests challenging hippocampus-dependent spatial working memory. APPΔS622 mice were impaired in the IntelliCage chaining task [54], as well as in the spontaneous T-maze alternation and in the eight-arm radial maze. Deficits in the T- and radial arm maze were observed in aged mice that exhibited a reduced overall spine density, while spine density was unchanged in young adult APPΔS622 mice. Together, this suggests an age-dependent worsening of the phenotype and may reflect an aging-dependent role of APP fragments for synapse maintenance and cognition. Interestingly, and consistent with our data, previous studies of healthy aging in wild-type rats reported an age-dependent decline of APPsα, weaker LTP expression, and deficits in object relocation that could be restored by recombinant APPsα [73, 74]. In addition to deficits in spatial working memory, APPΔS622 mice showed several behavioral changes that were not related to memory. In the serial reaction time task and during corner preference learning in the IntelliCage, they were less able than controls to suppress inappropriate nose pokes. In the open field test, they were less hesitant than controls to leave the protective wall zone. Given that APPΔS622 mice showed no evidence of general hyperactivity, neither in the conventional open field test nor in the IntelliCage where they were even slightly hypoactive, we interpret these behavioral changes as symptoms of mild motor impulsivity. Together, these observations suggest that physiological APP processing may have a broader role in cognition, not only supporting memory but also executive functions.

In summary, our findings significantly advance the understanding of APP physiology and reveal an essential role of endogenous APP processing for different forms of synaptic plasticity and cognition that are impaired in Alzheimer’s disease. As such, therapeutic approaches aimed at deleting APP or downregulating its expression [75–77] need to be carefully evaluated to avoid compromising essential endogenous functions of APP and its cleavage products for brain physiology and cognition.

## Materials and methods

### Mice

APPΔS622 mice express a deletion variant of APP lacking the α- and β-secretase cleavage site (ΔS592-G621, according to APP695 numbering) and were generated in collaboration with Ozgene (Perth, Australia) as described in more detail in Figure S1.

Mice were genotyped by a three-primer PCR using APPΔS622_01 (5’-GTC TTT TTG TTT GCT AGG TGG TGG-3’), APPΔS622_02 (5’-CAT GTC CGA ATT CTG CAT CCA TC-3’) and APPΔS622_04 (5’-TAC CAC AGA GGG ATG TTG CTT TTC-3’). Primer combination APPΔS622_01/APPΔS622_02 amplifies a 253 bp wild-type fragment, and APPΔS622_01/APPΔS622_04 a 501 bp fragment specific for the APPΔS622 knockin allele. Experiments involving animals were performed in accordance with the guidelines and regulations set forth by the German Animal Welfare Act and the Regierungspräsidium Karlsruhe, Germany. All the animals used in this study were chosen randomly. Animals were housed in the same room with a 12-h/12-h light/dark cycle in Makrolon Type II (360 cm^2^) cages with standard bedding, either alone or in groups, and had *ad libitum* access to standard chow and water.

### Expression analysis

#### RT-qPCR

For analysis of APP gene expression, mice were sacrificed by cervical dislocation. After removal of the brain, 25 mg of brain tissue was homogenized in 450 μl TRIzol reagent using two ceramic beads and a bead ruptor (Omni Bead Ruptor 24, Omni International, 2 × 20 s at 3.1 m/s). Total RNA was extracted using the Direct-zol RNA Miniprep Plus Kit (Zymo Research) according to the manufacturer’s instructions, including on-column DNase digestion. RNA was eluted in 50 μl DNase/RNase-free water. RNA concentration and purity were determined in triplicate using a NanoDrop spectrophotometer, and samples were adjusted to a final concentration of 0.1 μg/μl.

RT–qPCR was performed using the Luna® Universal One-Step RT–qPCR Kit (New England Biolabs) on a LightCycler® 96 system (Roche Diagnostics). The following primer pairs were used: APP_qPCR_for (5′-AGA AAG CTG CTC AGA TCC G-3′; binding within exon 12) and APP_qPCR_rev (5′-GTC TTC CTC GTC TTG ATG AGG-3′; binding within exon 14), yielding a 158-bp amplicon; GAPDH_for (5′-CGT CCC GTA GAC AAA ATG GT-3′) and GAPDH_rev (5′-TTG ATG GCA ACA ATC TCC AC-3′). Cycling conditions were as follows: reverse transcription at 55 °C for 10 min (1 cycle), initial denaturation at 95 °C for 1 min (1 cycle), followed by 45 cycles of denaturation at 95 °C for 10 s and extension at 62 °C for 30 s. A melting curve analysis (60–95 °C) was performed to confirm amplification specificity using the Light

Cycler® 96 Application Software (Roche Diagnostics).

Relative gene expression levels were calculated using the ΔΔCt method with GAPDH as an internal reference control.

#### Analysis of APP processing

Mice were euthanized by CO₂ inhalation at 3–4 months of age. Brains were rapidly removed, the cerebral hemispheres were separated, snap-frozen in liquid nitrogen, and stored at −80 °C until further use.

Frozen hemispheres were homogenized in 700 μl ice-cold tissue homogenization buffer (20 mM Tris-HCl, pH 7.4, 250 mM sucrose, 1 mM EDTA, 1 mM EGTA) supplemented with a protease and phosphatase inhibitor cocktail (Roche). Homogenization was performed using a bead ruptor (Omni Bead Ruptor 24, Omni International; 2 × 20 s at 3.1 m/s). Lysates were cleared by low-speed centrifugation (5000 × *g*, 5 min, 4 °C) to remove residual debris.

For detection of soluble APP fragments (total APPs, APPsα and APPsβ), cleared homogenates were subjected to ultracentrifugation (98,000 × *g*, 80 min, 4 °C). The resulting membrane-free supernatant was collected and used for further analyses. Total brain homogenate or supernatant (20 μg protein per lane) was resolved by SDS–PAGE on 12% Tris–glycine gels.

For analysis of APP C-terminal fragments (APP-CTFs), membrane pellets obtained after ultracentrifugation (UC: 98,000 × *g*, 80 min, 4 °C) were resuspended in Tris-buffered saline (10 mM Tris-HCl, 154 mM NaCl, pH 7.4). Proteins (20 μg protein per lane) were separated on 14% Tris–tricine gels.

Proteins were transferred onto Amersham™ Hybond™ P PVDF Membrane (0.45 μm; Cytiva) using a wet tank transfer system (400 mA, 45 min). For detection of APP-CTFs, proteins were transferred onto Amersham™ Protran™ Nitrocellulose Membrane (0.2 µm) with the Trans-Blot® Turbo™ semi-dry Transfer System (Bio-Rad). Membranes were blocked for 1 h at room temperature in 5% (w/v) nonfat dry milk in PBS containing 0.1% Tween-20 (PBS-T) and incubated with primary antibodies overnight at 4 °C. The following antibodies were used: Y188 (1:1000; Abcam, #ab21316) for full-length APP and APP-CTFs; 22C11 (1:1000; Millipore, #MAB348) for soluble APP; M3.2 (1:1000; kind gift from P. Mathews) and Mrk61 (1:1000; provided by Matthew Kennedy, Merck) [78] for detection of APPsα and APPsβ neo-epitopes; and GAPDH (1:2000; Merck, #ABS16) as loading control. For the detection of APLP1 and APLP2 in total brain homogenates, anti-APLP1 (1:1000, Synaptic Systems, #489 011) and anti-APLP2 antibodies (1:1,000, Synaptic Systems, #490 011) were used.

After washing in PBS-T, membranes were incubated with horseradish peroxidase-conjugated secondary antibodies (goat anti-mouse HRP, 1:10,000, Dianova, #1151-165-146; donkey anti-rabbit HRP, 1:10,000, Dianova, #711-035-152), washed again, and developed using enhanced chemiluminescence reagents (SignalFire ECL, #6883, or SignalFire Plus ECL, #12630; Cell Signaling Technology). Signals were detected and quantified using Image Lab software (Bio-Rad) and normalized to total protein per lane determined from stain-free membrane imaging [79]. Data were analyzed using unpaired Student’s *t*-test.

#### Measurement of Aβ

Following homogenization of one cerebral hemisphere in tissue homogenization buffer, the supernatant was collected and concentrated about eightfold using Amicon Ultra-0.5 centrifugal filter units (Millipore) according to the manufacturer’s instructions. Subsequently, 25 μl of each concentrated sample was loaded in duplicate onto a V-PLEX Aβ Peptide Panel 1 (4G8), Multi-Spot 96-well plate (Meso Scale Discovery; catalog no. #K15199G). The electrochemiluminescence immunoassay was performed according to the manufacturer’s protocol.

Aβ40 concentrations were normalized to total protein determined from the corresponding homogenates. Statistical analysis was performed using the Mann–Whitney U test. Cohort sizes were as follows: WT (*n* = 5) and APPΔS622 (*n* = 5). Mice were 4–5 months of age at the time of analysis.

#### Subcellular fractionation and western blot

Mice were euthanized by CO₂ inhalation, and both cortices were rapidly dissected and processed for subcellular fractionation as previously described [80, 81]. Tissue was homogenized using a Teflon–glass douncer (12 strokes at 900 rpm) in ice-cold homogenization buffer containing 10 mM HEPES (pH 7.4), 2 mM EDTA, 5 mM sodium orthovanadate, 30 mM sodium fluoride, 20 mM β-glycerophosphate, and protease inhibitor cocktail (Roche). The resulting homogenate was centrifuged at 500 × *g* for 5 min at 4 °C to remove nuclei and debris. The supernatant (S1 fraction) was subsequently centrifuged at 10,000 × *g* for 15 min at 4 °C to obtain a crude synaptosomal pellet (P2 fraction) and a cytosolic supernatant (S2 fraction).

The P2 pellet was resuspended in buffer containing 50 mM HEPES (pH 7.4), 2 mM EDTA, 2 mM EGTA, 5 mM sodium orthovanadate, 30 mM sodium fluoride, 20 mM β-glycerophosphate, 1% Triton X-100, and protease inhibitor cocktail (Roche), followed by centrifugation at 20,000 × *g* for 80 min at 4 °C. This step yielded a postsynaptic density-enriched pellet (P3 fraction) and a presynaptic, synaptic vesicle-enriched supernatant (S3 fraction). The P3 pellet was resuspended in 50 μl buffer containing 50 mM Tris (pH 9.0), 5 mM sodium orthovanadate, 30 mM sodium fluoride, 20 mM β-glycerophosphate, 1% sodium deoxycholate, and protease inhibitor cocktail (Roche). All fractions were snap-frozen in liquid nitrogen and stored at −80 °C until further analysis.

For immunoblot analysis, equal amounts of protein were separated by SDS–PAGE on 8% polyacrylamide gels and transferred onto Amersham™ Hybond™ P 0.45 μm PVDF membranes (Cytiva). Membranes were blocked for 1 h at room temperature in 5% (w/v) nonfat dry milk in PBST (PBS containing 0.2% Tween-20) and incubated overnight at 4 °C with primary antibodies. The following primary antibodies were used: Y188 (1:1,000; Abcam, #ab21316), β-tubulin (1:1000; Merck Millipore, #MAB3408), postsynaptic density protein-95 (PSD95; 1:1000; Merck Millipore, #MAB1598), Homer1 (1:1,000; Synaptic Systems, #160 023), choline acetyltransferase (ChAT; 1:1,000; Proteintech, #20747-1-AP), synaptophysin (1:1,000; Synaptic Systems, #101 002), and β-actin (1:1000; Santa Cruz Biotechnology). After washing three times with PBST, membranes were incubated for 1 h at room temperature with horseradish peroxidase (HRP)-conjugated secondary antibodies (goat anti-mouse HRP, 1:10,000, Dianova, 1#151-165-146; donkey anti-rabbit HRP, 1:10,000, Dianova, #711-035-152). Following three additional washes in PBST, signals were visualized using enhanced chemiluminescence reagents (SignalFire ECL, #6883, or SignalFire Plus ECL, #12630; Cell Signaling Technology). Densitometric quantification of Western blot bands was normalized to total protein determined from stain-free membrane imaging. Data were analyzed using an unpaired Student’s *t*-test.

### Culture of primary cortical neurons

Timed pregnancies were established by monitoring vaginal plug formation following mating. The day of plug detection was designated embryonic day (E) 0.5. Pregnant females were sacrificed at E15 or E16 by intraperitoneal injection of ketamine/xylazine (120 mg/kg ketamine and 20 mg/kg xylazine per body weight). Embryos were rapidly removed and placed in ice-cold HBSS supplemented with 100 mM HEPES (Gibco). Embryos were decapitated, and heads were transferred to dissection medium on a precooled metal block. Brains were carefully isolated from the skull, and meninges were removed under a stereomicroscope using fine forceps. Cerebral hemispheres were separated, and cortices were dissected and pooled.

For enzymatic dissociation, cortices were incubated in 0.5 ml HBSS/HEPES containing 0.05% trypsin (1:50 dilution of 2.5% trypsin solution, Invitrogen, Thermo Fisher Scientific, #15090046) per hemisphere at 37 °C for 20 min with gentle inversion every 3 min. The trypsin solution was removed, and tissue was washed three times with 5 ml DMEM supplemented with 2% fetal calf serum (FCS) to terminate enzymatic digestion. Cortices were then resuspended in 1 ml culture medium (Neurobasal medium (Gibco#21103049) supplemented with 2 mM L-Glumatine, 2% B27 (Gibco #17504044) and 2.500 U Penicillin/Streptomycin) and were mechanically dissociated by 8–10 gentle trituration steps using a fire-polished, prewarmed glass pipette. The cell suspension was adjusted by adding 0.5 ml culture medium per hemisphere and filtered through a 100 μm cell strainer (Sarstedt, #83.3945.100). For immunocytochemistry, glass coverslips were pre-coated with 500 μl poly-L-lysine hydrobromide (PLL; Sigma, #P2636; 1:200 dilution in borate buffer) overnight at 37 °C and 5% CO_2_. Coverslips were subsequently washed three times with sterile Milli-Q water, covered with 0.5 ml sterile Milli-Q water, and maintained in the incubator until cell seeding.

Cells were counted and plated at a density of 1.5 × 10^5^ neurons per well in a 24-well plate onto PLL–coated coverslips for immunocytochemistry, or at a density of 2 × 10^6^ neurons per well in 2 ml culture medium in a 6-well plate for cell surface biotinylation experiments.

A complete medium change was performed at 1 day in vitro (DIV1). From DIV7 onwards, half-medium changes were carried out every 4 days by replacing 50% of the conditioned medium with fresh culture medium.

To prevent overgrowth of proliferating non-neuronal cells, primary neurons were treated at DIV3 with 3 μM cytosine β-D-arabinofuranoside (AraC; Sigma-Aldrich, #C6645; 100 mM stock solution) added in 100 μl of additional culture medium.

#### Immunocytochemistry

Cells were washed once with Dulbecco’s Phosphate-Buffered Saline (DPBS) and fixed with 4% paraformaldehyde (PFA; 500 μl per well in a 24-well plate) for 15 min at room temperature. After two washes with DPBS, cells were permeabilized with 0.1% Triton X-100 in PBS (500 μl per well) for 10 min, followed by two additional washes with DPBS. Non-specific binding sites were blocked by incubation with 5% normal goat serum (NGS) in DPBS (300 μl per well) for 1 h at room temperature.

Primary antibodies were diluted in 5% NGS in DPBS and applied to each well (200 μl per well) for 1 h at room temperature under gentle agitation. The following primary antibodies were used: Y188 (1:500; Abcam) and MAP2 (1:1000; Synaptic Systems, #188 004). After four washes with PBS, cells were incubated with secondary antibodies diluted in blocking solution (200 μl per well) for 45 min at room temperature. The following secondary antibodies were used: Alexa Fluor 555–conjugated anti-guinea pig (1:500; Invitrogen, #A-21435) and Alexa Fluor 488–conjugated anti-rabbit (1:500; Invitrogen, # A32731). Cells were subsequently washed with PBS, incubated with DAPI (1:4000 in PBS), and subjected to two final washes with DPBS. Coverslips were mounted onto glass slides using Mowiol mounting medium.

Images were acquired using a Ti2 laser-scanning confocal microscope (Nikon). Image analysis was performed using FIJI (National Institutes of Health). Fluorescence intensity was quantified with strictly uniform laser settings as the mean gray value within a 50 μm radius surrounding the neuronal soma (*n* = 9 neurons per genotype). Background fluorescence was determined from a cell-free region and subtracted from each measurement. Data are presented in arbitrary units.

#### Metabolic glycoprotein labeling and biotinylation of cell surface glycoproteins

Primary cortical neurons were subjected to metabolic labeling at DIV 12 by supplementation of the culture medium with 50 μM Click-iT™ ManNAz (tetraacetylated N-azidoacetyl-D-mannosamine; Thermo Fisher Scientific, #C33366). Cells were incubated for 48 h at 37 °C and 5% CO₂. Control cultures received an equivalent volume of DMSO (vehicle control) to assess potential solvent-related effects.

At DIV14, cells were washed with ice-cold PBS (Thermo Fisher Scientific, #10010023) and subjected to click-chemistry–mediated biotinylation by incubation with 10 mM sulfo-dibenzocyclooctyne (DBCO)-biotin conjugate (Sigma-Aldrich, #760706) in 2 ml PBS per well for 1.5 h at 4 °C on a horizontal shaker. Following labeling, cells were lysed in ice-cold lysis buffer (150 mM NaCl, 2 mM EDTA, pH 8.0, 50 mM Tris-HCl, pH 7.5, 1% Triton X-100), using 150 μl per well for streptavidin pulldown experiments. Lysis was performed for 20 min at 4 °C under agitation (190 rpm), after which cells were scraped off, transferred to 1.5 ml low protein-binding tubes (Thermo Fisher Scientific, #90410), and centrifuged at 14,000 × *g* for 10 min at 4°C. Supernatants were collected, transferred to fresh tubes, and stored at −80 °C until further analysis.

#### Streptavidin pulldown of biotinylated cell surface proteins

For streptavidin pulldown experiments, 50 μl of Pierce™ streptavidin magnetic beads (Thermo Scientific, #88816) were transferred to 1.5 ml low protein-binding tubes (Thermo Fisher Scientific, #90410). Beads were washed twice with 500 μl Tris-buffered saline (TBS) containing 0.1% Tween-20 to remove storage buffer.

Neuronal cell lysates (136 μg total protein) were added to the equilibrated beads and incubated overnight at 4 °C under gentle agitation to allow binding of biotinylated proteins. Following incubation, beads were washed three times with 500 μl TBS containing 0.1% Tween-20 to remove non-specifically bound proteins.

Bound proteins were eluted by incubation with 80 μl Laemmli sample buffer supplemented with biotin at 95 °C for 5 min. Eluates (15 μl per lane) were subsequently resolved by SDS–PAGE. Densitometric quantification of Western blot bands was normalized relative to those from WT.

#### Detection of soluble APP in supernatant of neuronal cultures

To detect soluble APP species in the medium of primary cortical neurons, conditioned medium was collected from each well of a 6-well plate at DIV8. For concentration of secreted proteins, 2 ml of medium per sample was subjected to ultrafiltration using Amicon® Ultra centrifugal filter units with a 30 kDa molecular weight cutoff (Millipore, #UFC203024). Samples were centrifuged at 4000 × *g* for 15 min at 4 °C, and concentrated material was recovered by an additional centrifugation step at 1000 × *g* for 2 min.

Subsequently, 2.5 μl of the concentrated sample was loaded per lane and resolved by SDS–PAGE. Protein loading was normalized to the total protein amount per lane using stain-free technology, excluding the albumin band from the quantification.

### Spine density and morphology analysis

#### Iontophoretic fillings

For morphological analysis, CA1 pyramidal neurons were intracellularly filled via patch pipette with a solution containing 0.1–0.5% biocytin (Sigma-Aldrich). Acute slices were fixed in 4% Histofix (Carl Roth) and stored for 2–10 days. Slices were subsequently washed three times for 10 min each in 1× PBS and permeabilized for 1 h in 0.2% Triton X-100 in PBS (PBST). Biocytin-filled neurons were visualized by overnight incubation with Alexa Fluor 594–conjugated streptavidin (Life Technologies). On the following day, slices were washed three times for 10 min in PBS, air-dried at room temperature for 1 h, and mounted on coverslips using ProLong Gold Antifade mounting medium (Life Technologies).

#### Golgi staining

Golgi-Cox staining was performed using the Rapid Golgi Staining Kit (FD NeuroTechnologies, USA) following the manufacturer’s instructions. All procedures were conducted under dark conditions to protect the samples. One hemisphere from each mouse was immersed in 2.5 mL of an equal mixture of Kit Solutions A and B and incubated at room temperature for 2 weeks, with the solution refreshed after the first 24 hours. Following this incubation, brain tissues were transferred to Solution C and stored at 4 °C for a minimum of 72 h, with the solution replaced after 24 hours. Brains were then snap-frozen on dry ice, and 100 µm coronal sections were cut using a cryotome (Hyrax C50, Zeiss). Sections were mounted on adhesive microscope slides pre-coated with 1% gelatin and 0.1% chrome alum on both sides using Solution C. Staining was completed according to the manufacturer’s protocol, with the modification that RotiClear (Roth, Germany) was used in place of xylene. Finally, sections were cover-slipped with Permount mounting medium (Fisher Chemical, #225008).

#### Image acquisition

For spine density analysis, biocytin-filled or Golgi-stained neurons were imaged using an Axio Observer Z1 inverted fluorescence microscope (Zeiss) equipped with a Plan-Apo 63×/1.4 oil DICII objective (z-step size: 0.13 µm). For basal dendritic spine density, at least three segments of the basal dendritic tree were acquired per neuron. Segments were required to (i) run predominantly parallel to the slice surface, (ii) be located ≥ 20 μm from the soma, and (iii) display comparable shaft thickness. A minimum total basal dendritic length of 100 μm per neuron was analyzed. For midapical spine density, at least three segments from the middle third of the apical dendrite—defined as the central third between the origin at the soma and the distal tuft—were imaged. Selected segments had to (i) be of second or third order, (ii) lie within the midapical region, and (iii) exceed 10 μm in length. A minimum of 100 μm midapical dendritic length per neuron was evaluated.

Image files (zvi format) were imported into ImageJ (NIH) using the Bio-Formats importer and saved as TIFF files for analysis. Dendritic spines were manually counted by an experimenter blind to genotype using Neurolucida and NeuroExplorer software (MicroBrightField), according to established criteria [82] with minor modifications: (i) protrusions extending laterally from the dendritic shaft and exceeding 0.4 μm in length were counted as spines; (ii) spines projecting into the z-plane were included only if they extended > 0.4 μm laterally beyond the shaft; (iii) bifurcated protrusions were counted as two spines; and (iv) spines located within 10 μm of branch points or the soma were excluded. Spine density was expressed as spines per μm dendritic length. Data acquisition and analysis were performed blind to genotype.

For spine morphology analysis, dendritic segments were imaged using a Ti2 laser-scanning confocal microscope (Nikon) equipped with a 60× oil-immersion objective (5× scan zoom; 1024 × 512-pixel resolution; z-step size 0.25 μm). Imaging parameters were optimized individually to maximize signal without saturation. Segments were included if they were ≥ 30 μm in length, uniformly filled, and displayed sufficient signal-to-noise ratio for deconvolution. Confocal stacks were deconvolved using Huygens Professional (v3.8; Scientific Volume Imaging). Two to three basal and midapical segments per neuron of ≥ 100 µm dendritic length in sum with optimal post-deconvolution quality were selected for analysis. Image processing and quantitative analysis were performed in FIJI, with investigators blinded to genotype.

For spine head size measurements, image brightness was adjusted such that 0.1% of shaft pixels were saturated. The maximal cross-sectional spine head area was determined by manually outlining spine heads using a consistent gray-value threshold across segments with the SpineAnalyzerJ plugin (kindly provided by Dr. Tassilo Jungenitz). Only spines with a clearly discernible head were included in the analysis.

### Electrophysiology

#### Extracellular field recordings

Extracellular field recordings were performed in acute hippocampal slices obtained from 4–5-month-old APPΔS622 and wild-type littermate (LM) mice (*N* = 5–6 animals per genotype). Animals were group-housed under standard conditions (12 h light/dark cycle, controlled temperature and humidity) with food and water available ad libitum. All experiments were conducted blinded to genotype.

#### Acute hippocampal slice preparation and recording

Transverse hippocampal slices (400 μm) were prepared from isoflurane-anesthetized mice. Following decapitation, brains were rapidly removed and transferred into ice-cold, carbogenated (95% O₂/5% CO₂) artificial cerebrospinal fluid (ACSF) containing 125 mM NaCl, 2 mM KCl, 1.25 mM NaH₂PO₄, 2 mM MgCl₂, 26 mM NaHCO₃, 2 mM CaCl₂, and 25 mM glucose. Slices were cut using a vibrating microtome (VT1200S, Leica) and allowed to recover in carbogenated ACSF at room temperature for at least 1.5 h.

For recordings, slices were transferred to a submerged chamber perfused with carbogenated ACSF (32 °C; 1.2-1.5 ml/min). Field excitatory postsynaptic potentials (fEPSPs) were recorded from the *stratum radiatum* of the CA1 region using borosilicate glass electrodes (2–4 MΩ) filled with 3 M NaCl at a depth of 150–200 μm. Schaffer collaterals were stimulated with monopolar tungsten electrodes at 0.1 Hz. Baseline responses were recorded for 20 min at 40% of the maximal fEPSP slope. Long-term potentiation (LTP) was induced using theta-burst stimulation (TBS; 10 trains of four pulses at 100 Hz, 200-ms inter-train interval, repeated three times).

Basal synaptic transmission was assessed by input–output (IO) analysis using either defined stimulus intensities (25–250 μA) or fiber volley amplitudes (0.1–0.8 mV). Short-term plasticity was examined by paired-pulse facilitation (PPF) using interstimulus intervals ranging from 10 to 160 ms.

#### Rescue experiments with recombinant APPsα

For rescue experiments, acute hippocampal slices from wild-type LM and APPΔS622 mice were preincubated for 1 h at room temperature in 10 ml carbogenated ACSF containing 10 nM recombinant APPsα (recAPPsα). Recombinant APPsα was purified from the supernatant of stably transfected HEK cells as previously described [43, 44]. During recordings, slices were continuously perfused in a closed-loop system containing 50 ml ACSF supplemented with 10 nM recAPPsα.

#### Data analysis and statistics of extracellular field recordings

Electrophysiological data were acquired and analyzed using LabVIEW (National Instruments). The initial slope of fEPSPs was measured, normalized to baseline, and expressed as mean ± SEM. PPF was calculated as the ratio of the second to the first fEPSP slope multiplied by 100. Statistical analyses were performed using Microsoft Excel or GraphPad Prism. Comparisons between genotypes were conducted using the two-tailed Mann-Whitney test, whereas comparisons between recAPPsα-treated and untreated slices were analyzed using the one-tailed Mann-Whitney test. Differences were considered statistically significant at *p* < 0.05 (*p < 0.05, **p < 0.01, ***p < 0.001).

#### Organotypic slice culture preparation

Organotypic slice cultures (300 μm thickness) encompassing the ventral hippocampus and entorhinal cortex were generated from horizontal brain sections obtained from postnatal day 3–5 from mice of either sex (*n* = 1-2 cultures per *N* = 3-4 mice), following previously established protocols [83]. Cultures were maintained at 35 °C in a humidified incubator with 5% CO₂ and allowed to mature for at least 18 days prior to experimental use. The culture medium was refreshed three times per week and consisted of 50% (v/v) minimum essential medium (MEM), 25% (v/v) basal medium Eagle (BME), and 25% (v/v) heat-inactivated horse serum, supplemented with 25 mM HEPES, 0.15% (w/v) NaHCO₃, 0.65% (w/v) glucose, 0.1 mg/ml streptomycin, 100 U/ml penicillin, and 2 mM GlutaMAX. The final pH was adjusted to 7.3 using HCl or NaOH.

#### Whole-cell patch-clamp recordings

Whole-cell voltage-clamp recordings were obtained from CA1 pyramidal neurons in organotypic slice cultures at 35 °C (2–5 neurons per culture). The extracellular recording solution contained 126 mM NaCl, 2.5 mM KCl, 26 mM NaHCO₃, 1.25 mM NaH₂PO₄, 2 mM CaCl₂, 2 mM MgCl₂, and 10 mM glucose, and was saturated with 95% O₂/5% CO₂. Patch pipettes were filled with an internal solution comprising 126 mM K-gluconate, 4 mM KCl, 4 mM Mg-ATP, 0.3 mM Na₂-GTP, 10 mM phosphocreatine, 10 mM HEPES, and 0.1% (w/v) biocytin (pH 7.25 adjusted with KOH; 290 mOsm adjusted with sucrose).

For the isolation of AMPAR-mediated mEPSCs, organotypic tissue cultures were incubated with 0.5 μΜ ΤΤΧ, 10 μΜ ΑPV, and 10 μΜ Bicuculline methiodide. Recordings were performed at a holding potential of −70 mV. Signals were acquired at a sampling rate of 10 kHz with a 6 kHz low-pass Bessel filter. Series resistance was monitored in 2–4 min intervals, and recordings were discarded if the series resistance reached ≥30 MΩ and the leak current changed significantly. To assess whether Aβ restores homeostatic synaptic plasticity in CA1 pyramidal neurons [52], synthetic Aβ_1-40_ (1.5 μM; Bachem, #4035886) was applied in the presence of TTX, and cells were voltage-clamped at −70 mV. For Aβ preparation, lyophilized peptide was dissolved in 0.01 M PBS, vortexed three times for 1 min on ice, aliquoted, and stored at −80 °C. Prior to application, Aβ_1-40_ aliquots were slowly thawed on ice and vortexed again before addition to the culture medium. AMPAR-mediated mEPSCs were digitized using a Digidata 1550B digitizer and analyzed using Clampfit (Molecular Devices), utilizing the template method. Data were analyzed using the Mann–Whitney test and, for multiple group comparisons, using the Kruskal–Wallis test with Dunn’s *post hoc* test. All experiments were conducted blinded to genotype.

### Behavioral analysis

#### IntelliCage testing

A total of 29 female mice were included in the IntelliCage (IC) experiments, comprising 13 wild-type littermates and 16 APPΔS622 mice. At the start of behavioral testing, animals were 7–9 months old. All procedures were approved by the Veterinary Office of the Canton of Zurich (license 60/2021). Data acquisition and analysis were conducted with the experimenter blinded to genotype.

The following IC paradigms were performed in sequence: exploration and free adaptation, nose poke adaptation I, serial reaction time task, nose poke adaptation II, drinking session adaptation I, corner preference task (acquisition and reversal), drinking session adaptation II, and chaining task. Up to 13 female mutant and control mice were housed and tested together in a single IntelliCage.

For individual identification, mice were implanted subcutaneously with RFID transponders (Datamars SA, Bedano, Switzerland). The IntelliCage system (NewBehavior AG, Zurich, Switzerland) was installed within a standard Tecniplast 2000P cage (20 cm height, 55 cm length, 38 cm width). The apparatus contains four operant conditioning chambers positioned in the cage corners, each occupying a triangular floor area (15 × 15 × 21 cm). Entry into each chamber occurs through a tubular antenna (50 mm outer, 30 mm inner diameter) that reads the transponder code.

Each chamber is equipped with a proximity sensor and two circular nose-poke openings (13 mm diameter; left and right), providing access to water bottle nipples. Nose pokes are detected via infrared photo beams, and access to water can be controlled by motorized doors. Four triangular red shelters (Tecniplast, Buguggiate, Italy) were placed in the center of the cage, serving as resting areas and as platforms to access food. The floor was covered with 2–3 cm of aspen bedding (5 × 5 × 1 mm; Abedd, Lab & Vet Service GmbH, Vienna, Austria). The system was operated by dedicated software that executed predefined schedules and recorded visits, visit duration, nose pokes, and licking behavior for each individual mouse.

#### Statistical analysis of IntelliCage testing

Statistical [84] analyses were performed using mixed-design analysis of variance (ANOVA) models with genotype (APPΔS622 vs. WT) as a between-subject factor. Within-subject factors were included as appropriate to assess effects of place, time, or stimulus.

Significant interactions and, where relevant, main effects were further examined using Bonferroni *post hoc* tests or by splitting the ANOVA model. One-sample *t*-tests were used for comparisons against chance levels. Variables exhibiting skewed distributions or frequent outliers (e.g., latency measures) were log(1+x)-transformed prior to analysis.

The significance threshold was set at *p* < 0.05. To account for multiple comparisons within conceptually related variable groups of a given test, the false discovery rate (FDR) procedure according to Hochberg was applied. As genotype and treatment effects were independent of sex, sex was not included as a factor in the reported results or figures. Statistical analyses were performed using R (version 3.2.3) with the packages psych and moments.

##### Adaptation phases

During the initial four days in the IntelliCage, mice underwent a free adaptation phase in which all corner doors remained open, allowing unrestricted access to all eight drinking bottles.

This was followed by a three-day nose-poke adaptation phase. During this period, all doors were kept closed and could only be opened by a nose poke, granting access to water for 5 s per visit.

Subsequently, mice were subjected to a drinking session adaptation phase, during which access to water was restricted to defined time windows. Doors opened in response to a nose poke only during four daily sessions (02:00–03:00, 11:00–12:00, 14:00–15:00, and 23:00–24:00). Outside these scheduled sessions, water was not available.

##### Serial Reaction time task

In this task, all four corners operated in the same way, 24 h per day: the first nose poke in a visit determined the correct side and initiated a delay period, after which, on the correct side and for a period of 5 s, 3 green LEDs were switched on and the door opened for drinking. Any nose poke during the delay period was considered a premature response, whereas the first nose poke at the open door was counted as a correct response. Correct response latency was defined as the time that elapsed between the onset of the light stimulus and the correct response. The task had three stages. During the first 3 days, delays were set at 0 s (baseline). Then, the delays varied randomly between 0.5, 1.5, and 2.5 s for the rest of the task. During these 4 days, premature responses had no consequence.

##### Corner preference learning and chaining tasks

In these paradigms, water was available in only one of the four IntelliCage corners during each drinking session. The rule determining the rewarded corner differed between tasks.

In the corner preference task, water was accessible in the same designated corner for four consecutive days (acquisition phase). To minimize social learning effects, cage mates were assigned to four subgroups, each with a different target corner.

Subsequently, mice were tested in the chaining task, in which the rewarded corner depended on the animal’s previous visit. Water was delivered in the corner adjacent to the most recently visited one in which at least one nose poke had been made, following a clockwise (or anticlockwise) sequence. Mice were trained for 6 days. Performance was calculated as the percentage of correct visits that included a nose poke, corrected for chance level (25%).

#### Non-IntelliCage behavioral testing of mice of both sexes

Neuromotor and cognitive performance were assessed in 32 mice, comprising 15 wild-type littermates (5 females, 10 males) and 17 APPΔS622 mice (8 females, 9 males). Animals were housed in groups of 2–5 under a 12 h light/dark cycle (lights on at 20:00) with food and water available ad libitum, unless single housing was required for experimental or welfare reasons. Behavioral testing was conducted during the dark phase under dim illumination (~22 lux). Mice were habituated to the testing room for 30 min prior to each session, and experimenters were blinded to genotype unless otherwise stated. All procedures were approved by the Veterinary Office of the Canton of Zurich (license 60/2021). Animals were 13–14 months old at the onset of testing. The test battery was administered over 9 weeks (including recovery periods) in the following order: open field, rotarod, grip strength, T-maze and radial maze. One female APPΔS622 mouse died after 5 weeks and did not complete all tests; no other animals were excluded.

##### Open field

Spontaneous locomotor activity and exploration were assessed in a circular open field (150 cm diameter) over two consecutive days (10 min/session), as previously described [85]. Behavior was recorded and analyzed using EthoVision XT17 (Noldus). The arena was subdivided into a wall zone (18% of total area; 7 cm width), a center zone (50%), and a transition zone. For analysis of stereotypic behavior, the arena was further divided into a 5 × 5 grid, and repeated tile-to-tile movement sequences were quantified. The stereotypy index was calculated as the percentage of repeated tile transitions relative to total crossings.

##### T-maze spontaneous alternation

Spontaneous alternation was assessed using a gray PVC T-maze (arms: 30 × 10 cm) as described [86]. A removable central partition (extending 7 cm into the start arm) and guillotine doors at goal arms were used to minimize extraneous cues. Each trial consisted of a sample run followed immediately by a choice run. During the sample phase, the mouse was confined to one goal arm for 30 s. For the choice phase, the partition was removed, and the mouse was allowed to choose freely. Alternation was defined as entry (whole body including tail) into the arm opposite to that visited during the sample run. Three trials were conducted per day (inter-trial interval ~60 min), for a total of six trials per animal. Performance was expressed as the percentage of correct alternations.

##### Radial arm maze

Working memory was evaluated using an 8-arm radial maze as previously described [57]. Arms (7 × 38 cm) extended from an octagonal central platform (diameter 18.5 cm) and were equipped with transparent Perspex walls (5 cm height). A single millet seed (~0.05 g total) was placed in a recessed cup at the end of each arm. Mice were food-restricted to 85% of their free-feeding body weight beginning two days prior to testing and maintained at this level throughout the experiment; water was available ad libitum.

Following habituation (10 min exposure to the baited maze), mice underwent one session per day (maximum 10 min) for 10 consecutive days. Sessions ended when all eight rewards were collected or after 10 min. Performance was video-tracked; first arm entries and reward retrieval were recorded manually. Outcome measures included the number of correct choices within the first eight entries, re-entry errors as a function of trial and rewards collected, and arm preference. In error-free performance, preferred arm visits corresponded to chance level (12.5%). Values between 12.5% and 25% indicated re-entries, whereas values > 25% reflected a strong arm preference.

#### Statistical analysis of non-IntelliCage behavioral testing

Behavioral data were analyzed using ANOVA with genotype (APPΔS622 vs. WT) as a between-subject factor. Within-subject factors were included where appropriate to assess effects of time, location, or stimulus. Variables exhibiting non-normal distributions or heteroscedasticity were subjected to Box–Cox transformation prior to analysis. Statistical significance was defined as *p* < 0.05.

## Supplementary information

Kilian_et_al_Supplementary_information

Kilian_et_al_Original_Western_Blots

## Data Availability

The datasets and materials generated and/or analyzed during the current study are available from the corresponding author upon reasonable request.
