# Supplementary material for: Physiological APP processing is required for synaptic plasticity and cognition
Source: Cell Death Dis. 2026 Nov 4;17(1):941. doi: 10.1038/s41419-026-09378-6 (PMC13737196; doi:10.1038/s41419-026-09378-6)

Original Western Blots

Figure 1E-J:

Bands indicated by red lines are depicted in Figure 1E. Bands indicated by the green boxes were used for quantification (Figure 1F-J).

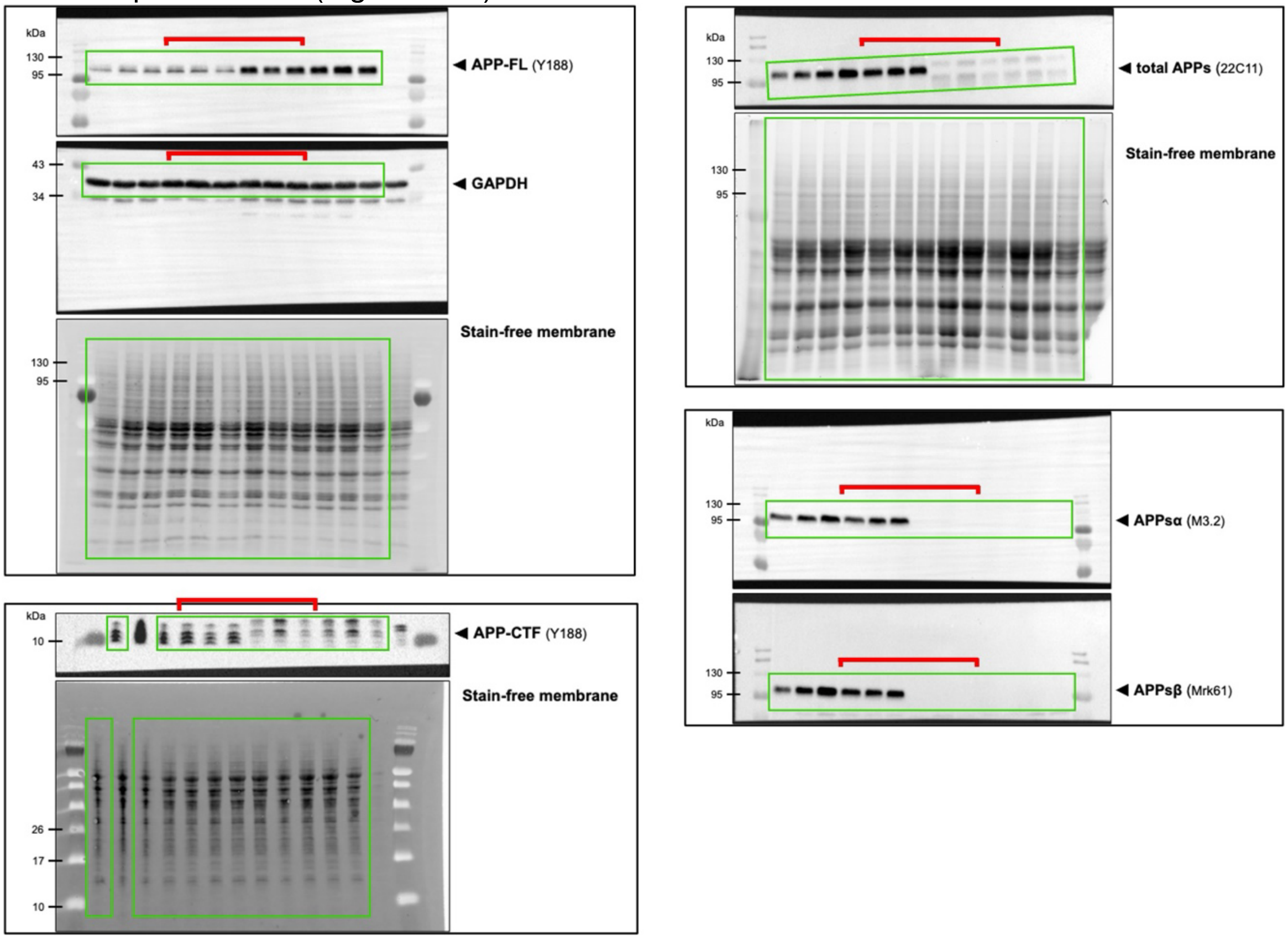

Figure 1L-N:

Bands indicated by red lines are depicted in Figure 1L. Bands indicated by the green boxes were used for quantification (Figure 1M-N).

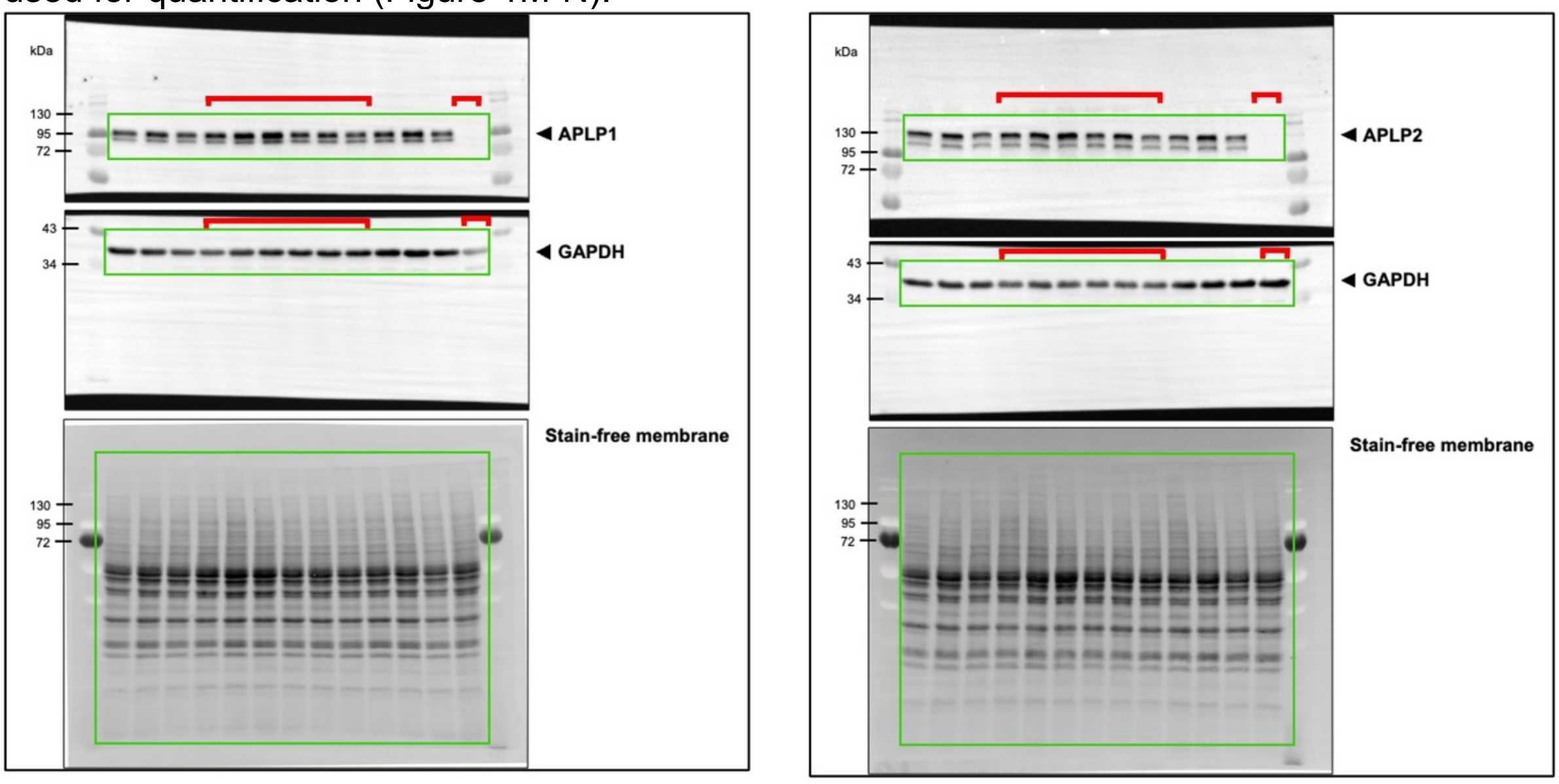

Figure 2C-E:

Bands indicated by red lines are depicted in Figure 2C. Bands indicated by the green boxes were used for quantification (Figure 2D-E).

Cell surface APP

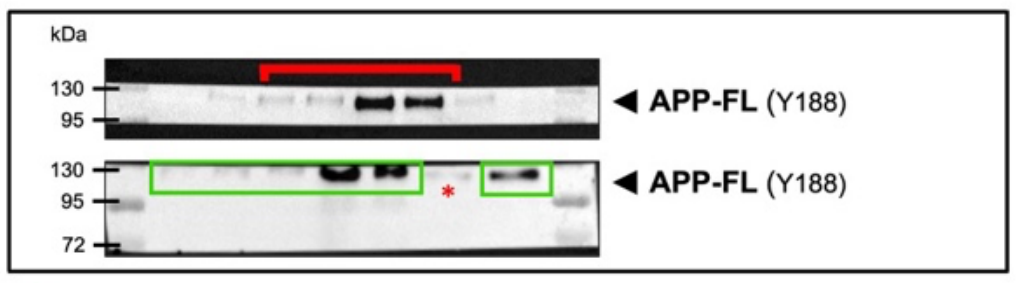

* Sample was excluded due to technical failure of the streptavidin pulldown.

Secreted APPs

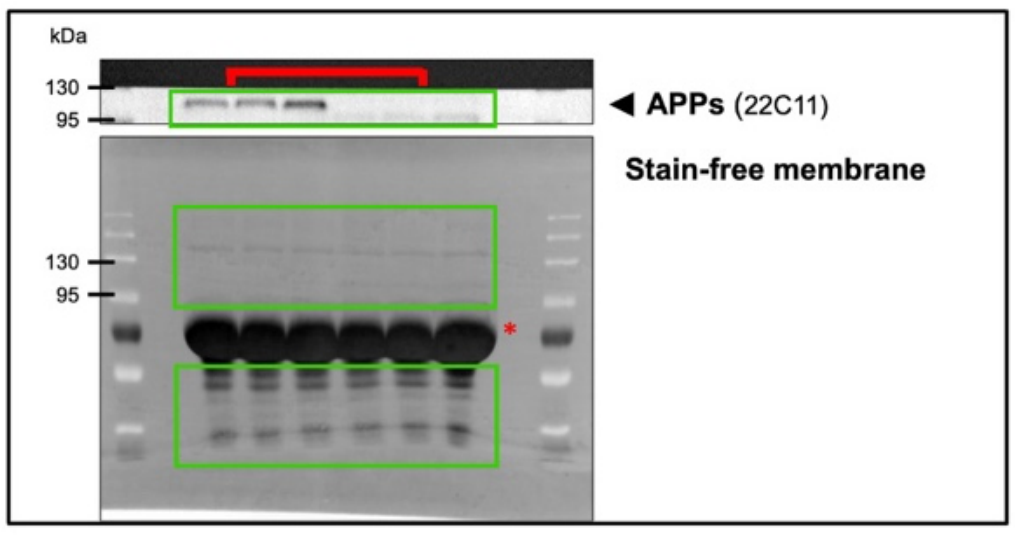

* Band corresponding to albumin, which is highly abundant in the neuronal medium, was excluded from normalization to the stain-free membrane signal.

Figure 2F:

Bands indicated by red lines are depicted in Figure 2F.

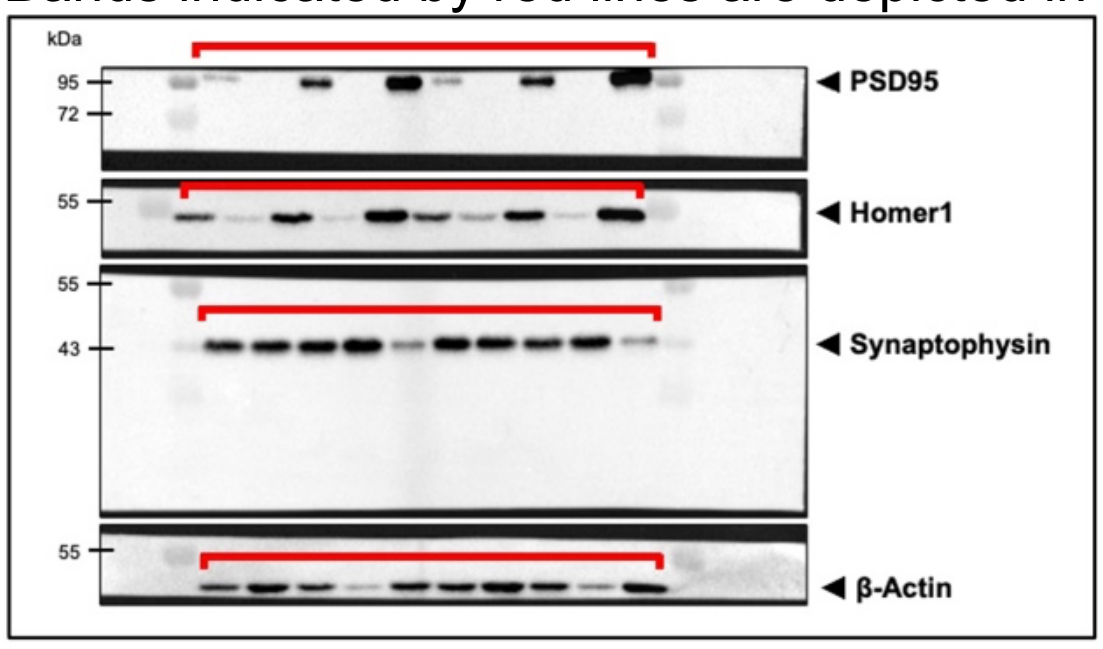

Figure 2G-I:

Bands indicated by red lines are depicted in Figure 2G. Bands indicated by the green boxes were used for quantification (Figure 2H-I).

Presynaptic, vesicle-rich fraction (S3)

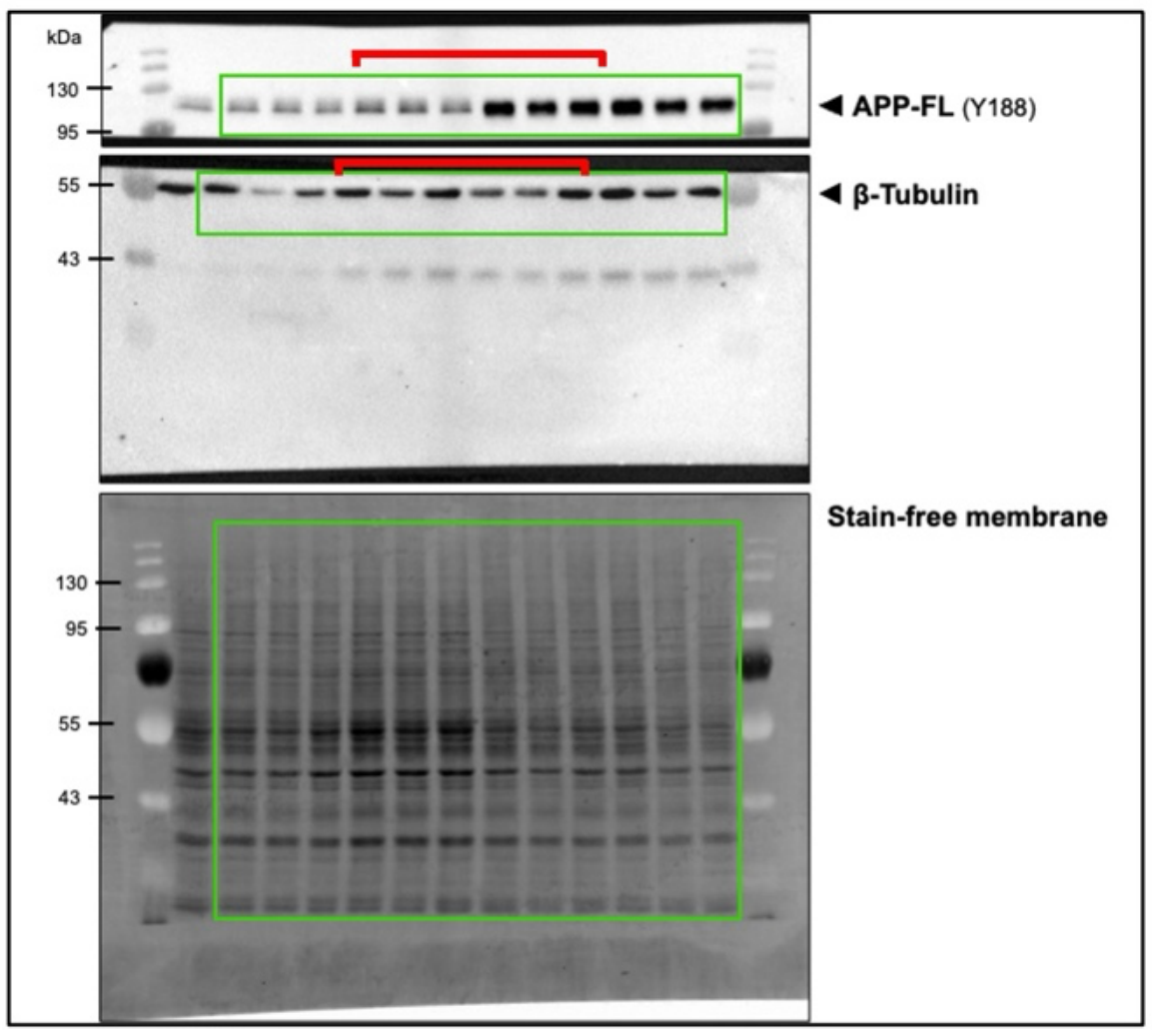

Postsynaptic density fraction (P3)

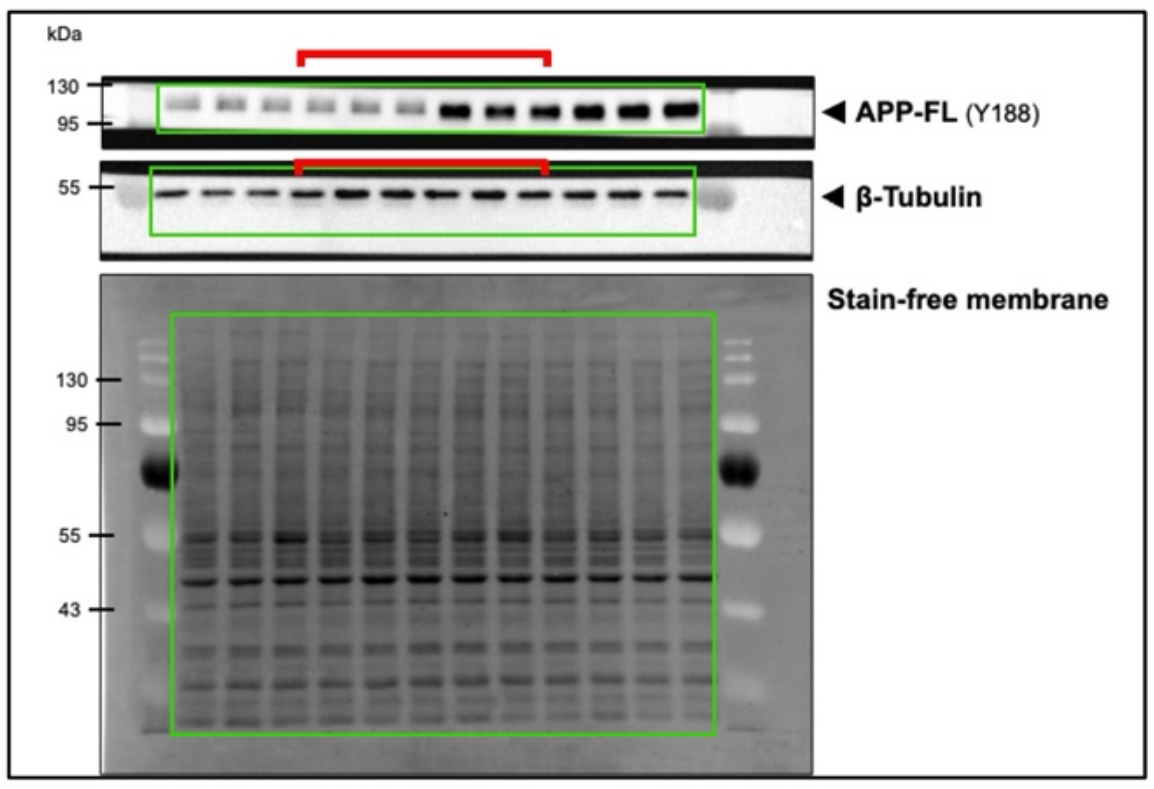

Supplement: Supplementary file 1 [file 41419_2026_9378_MOESM2_ESM.pdf]
