# Supplementary material for: Physiological APP processing is required for synaptic plasticity and cognition
Source: Cell Death Dis. 2026 Nov 4;17(1):941. doi: 10.1038/s41419-026-09378-6 (PMC13737196; doi:10.1038/s41419-026-09378-6)
Supplement: Supplementary file 2 [file 41419_2026_9378_MOESM1_ESM.docx]

Supplementary information

Physiological APP processing is required for synaptic plasticity and cognition

Lara Kilian, Verena Bengelsdorff, Jennifer Just, Sebastian Stork, Christos Galanis, Marija Banicevic, Irmgard Amrein, Michaela Back, Tassilo Jungenitz, Meike Fellenz, Jakob von Engelhardt, Thomas Deller, David P. Wolfer, Andreas Vlachos, Martin Korte, Ulrike C. Müller

**Inventory of Supplementary Material**

1. **Supplementary methods**

- Nissl staining
- Immunohistochemistry
  - Statistical analysis
- Stereology
- Neuronal morphology
- Motor behavior
  - Grip strength
  - Rotarod

2. **Supplementary figures**

- **Figure S1:** Gene targeting strategy for the generation of APPΔS622 knockin mice.
- **Figure S2:** Experimental design.
- **Figure S3:** Normal brain morphology of APPΔS622 knockin mice.
- **Figure S4:** Sholl analysis reveals unchanged dendritic complexity of CA1 pyramidal neurons in APPΔS622 mice.
- **Figure S5:** IntelliCage setup and behavioral testing paradigm.
- **Figure S6:** Normal motor strength and coordination in APPΔS622 mice.

3. **Supplementary references**

# Supplementary methods

## Nissl staining

Animals were euthanized by CO₂ inhalation and transcardially perfused with PBS, followed by fixation with 4% paraformaldehyde (PFA) in PBS for 5 min. Brains were carefully removed from the skull and postfixed overnight at 4°C in 4% PFA in PBS. Coronal sections (40 μm thickness) were prepared using a Vibratome (HM650V microtome) and collected in cold PBS in 24-well plates.

For Nissl staining, free-floating sections were incubated in 500 μl PBS per well supplemented with two drops of a toluidine blue/methylene blue staining solution for 10 min at room temperature. Sections were subsequently washed with 1× PBS to remove excess dye and mounted onto coverslips using Permount mounting medium (Fisher Chemical, #225008).

## Immunohistochemistry

Animals were euthanized by CO₂ inhalation and transcardially perfused with PBS, followed by fixation with 4% paraformaldehyde (PFA) in PBS for 5 min. Brains were carefully removed from the skull and postfixed overnight at 4°C in 4% PFA in PBS. Coronal sections (40 μm thickness) were prepared using a Vibratome (HM650V microtome) and collected in cold PBS in 24-well plates.

Free-floating sections were blocked and permeabilized overnight at 4°C in PBS containing 5% (w/v) bovine serum albumin (BSA), 5% (v/v) normal goat serum (NGS), and 0.4% Triton X-100. Sections were then incubated overnight at 4°C with primary antibodies diluted in PBS supplemented with 5% NGS and 0.2% Triton X-100 (300 μl per well). After washing with PBS, sections were incubated for 2 h at room temperature with appropriate secondary antibodies diluted in PBS containing 0.1% BSA and 0.05% Triton X-100 (300 μl per well).

Following additional washes, sections were stained with DAPI in PBS, mounted onto Superfrost microscope slides (Menzel), and coverslipped using Mowiol mounting medium.

The following primary antibodies were used: Iba1 (rabbit, 1:1,000, Synaptic Systems, #234 008), GFAP (mouse, 1:1,500, Invitrogen, #14-9892-82), NeuN (guinea pig, 1:1,500, Millipore, #ABN90), calbindin (rabbit, 1:3,000, Swant, #CB38), Ctip2 (rat, 1:250, Abcam, #25B6). Secondary antibodies included goat anti-rabbit Cy3 (1:1,500, Dianova, #111-165-144), goat anti-mouse Alexa Fluor 647 (1:1,500, Dianova, #115-605-062), goat anti-rat Alexa Fluor 488 (1:500, Jackson ImmunoResearch, #112-545-167), goat anti-rabbit Alexa Fluor 647 (1:1,500, Invitrogen, #A-21244 ) and goat anti-guinea pig Alexa Fluor 488 (1:500, Abcam, # ab150185). Images were acquired using an Axio Observer Z1 microscope (Zeiss). Exposure times were optimized using wild-type samples and subsequently maintained constant for APPΔS622 brain slices.

### Statistical analysis

For quantification of GFAP-, Iba1-, and NeuN-positive cells, a standardized rectangular region of interest (200 × 600 μm) was defined in the CA1 region of the hippocampus using ImageJ. The region was positioned distal to the apex of the *stratum pyramidale* with the pyramidal layer centered, and the fornix and *stratum pyramidale* were excluded using the freehand selection tool to restrict analysis to the surrounding hippocampal area.

GFAP-, Iba1-, and NeuN-positive cells were manually counted. DAPI-positive nuclei were quantified using the StarDist plugin in ImageJ following background subtraction (rolling ball radius: 3.0 pixels) and brightness/contrast adjustment (display range 8–29). For GFAP quantification, background subtraction (rolling ball radius: 3.0 pixels) and brightness/contrast adjustment (−1 to 77) were applied prior to manual counting, whereas Iba1 and NeuN channels were analyzed without further image adjustment.

For cortical layer analysis, images were rotated in ImageJ to align the cortex horizontally above the stratum granulosum. A rectangular region of interest (300 μm width) was defined within a standardized cortical area by drawing a line from the lower border of the stratum granulosum through the cortex and centering the rectangle along this axis. Individual cortical layers within the region of interest were delineated using the freehand selection tool, and their areas were measured. Layer height was calculated by dividing the measured area by the fixed rectangle width (300 μm). Data acquisition and analysis were performed blind to genotype.

## Stereology

Brain volume of five animals per genotype (age: 2-3 months, both sexes) was estimated in every tenth 20 μm coronal section of methacrylate-embedded hemispheres stained with Giemsa. Volumetric analysis was performed using the Cavalieri estimator with a 200 × 200 μm point grid. For inter-animal and inter-group comparisons, calculated volumes were allocated to 12 equidistant bins along the rostro-caudal axis of each brain, as previously described (1). The precision of volume estimates was determined using the Gundersen–Jensen coefficient of error (CE) with a conservative smoothness parameter (m = 0), providing an estimate of the expected variability upon repeated measurements (2,3).

## Neuronal morphology

For analysis of neuronal morphology, overview images of biocytin-filled CA1 pyramidal neurons were taken using an Axio Observer Z1 inverted fluorescence microscope (Zeiss) equipped with a 20× objective (z-step size: 0.5 μm). Basal and apical dendritic trees were imaged separately using two overlapping z-stacks per compartment. Exposure times were adjusted individually for each neuron to ensure optimal use of the dynamic grayscale range.

Neurons were manually reconstructed using Neurolucida software (MicroBrightField) by an experimenter blinded to genotype. Only neurons with a completely filled apical or basal dendritic tree with clearly defined terminal branches were included in the analysis. Sholl analysis was performed using NeuroExplorer software (MicroBrightField). Briefly, concentric spheres centered on the soma were generated at 30 μm intervals, and the number of dendritic intersections and dendritic length between successive spheres were quantified. Basal and apical dendrites were analyzed separately, and parameters were plotted as a function of distance from the soma. Data acquisition and analysis were performed blind to genotype.

## Motor behavior

### Grip strength

Forelimb grip strength was assessed as previously described (4) using a digital force gauge (maximum force: 300 g). Mice were allowed to grasp a horizontal metallic bar (4 cm length, 2.5 mm diameter) attached to the force meter with both forepaws while being gently held by the tail. The animal was then steadily pulled backward until it released the bar. Each mouse completed five trials per day over two consecutive days. The maximal force achieved in each trial was recorded, and mean peak force values were calculated for statistical analysis.

### Rotarod

Motor coordination and balance were evaluated using a Rotarod apparatus (Ugo Basile, model 47600, Comerio, Italy). The rotating drum accelerated linearly from 2 to 40 rounds per minute (rpm), reaching maximal speed after 290 s. Each mouse underwent five trials on a single day. Trials ended when the mouse fell from the rod or after a maximum duration of 300 s. The latency to first passive rotation (i.e., clinging to the rod and completing a full passive turn), as well as the latency to fall and corresponding rotational speed, were recorded. Mean values across trials were used for analysis.

# Supplementary figures

## Supplementary Figure S1. Gene targeting strategy for the generation of APPΔS622 knockin mice.

**(A)** Genomic mouse APP locus encompassing exon 15-18. Genotyping with the primers shown by green arrows produced a 253 bp fragment in wild-type animals.

**(B)** The gene targeting vector contained a minigene encoding the APP C-terminus (exon17-18 including the stop codon and 3´-UTR, followed by a rabbit β-globin polyA signal, rBGpA depicted in red) that was inserted behind exon 16. Further downstream, a hybrid exon 16/17 (grey/blue) encoding the APPΔS622 mutation was introduced. Hygromycin and neomycin cassettes (light grey) were inserted to allow for positive selection of ES cells that underwent homologous recombination. In addition, loxP sites (red flags) were inserted upstream of exon 16 and upstream of the hybrid exon 16/17, allowing for either conditional expression of the APPΔS622 variant or removal of the loxP-flanked region to generate constitutive APPΔS622 mice that were used in this study. The targeting vector also contained flanking regions of homology that are not drawn to scale. Left arm of homology: 3.01 kb; right arm of homology: 3.06 kb.

**(C)** Targeted allele after homologous recombination in ES cells.

**(D)** Constitutive APPΔS622 knockin allele after Cre-mediated deletion of the floxed genomic region using ROSA26-Cre deleter mice. Note that splicing (depicted by a triangle) will connect the hybrid exon 16/17 containing the APPΔS622 deletion to endogenous exon 18 harboring the stop codon. Homozygous mice of this genotype were used for all experiments of this study. Genotyping with the primers shown by green arrows produced a 501 bp fragment in APPΔS622 animals.

### Supplementary text to Figure S1:

APPΔS622 mice were generated by gene targeting using Bruce4 C57BL/6 ES cells (Ozgene) in collaboration with Ozgene (Perth, Australia). Correctly targeted ES cells were injected into goGermline blastocysts (5), and resulting chimeras were subsequently crossed to ROSA26-targeted Cre-deleter mice (Gt(ROSA)26Sor^tm1(PGK1-cre)Ozg^) to constitutively remove the loxP-flanked genomic region in early embryos. Heterozygous APPΔS622 knockin mice were bred to homozygosity, and wild-type littermates were used as internal controls. All mice were of pure C57BL/6 genetic background.

## Supplementary Figure S2. Experimental design.

|  | **Method** | | **Age [months]** | **Sex** | **Number of animals (WT)** | **Number of animals (APPΔS622)** |
| --- | --- | --- | --- | --- | --- | --- |
| **APP expression and processing analysis** | RT-qPCR |  | 3-4 | mixed | 6 | 6 |
|  | Western Blot | APP-FL | 3-4 | mixed | 12 | 12 |
|  |  | APPs | 3-4 | mixed | 12 | 12 |
|  |  | APP-CTF | 3-4 | mixed | 6 | 5 |
|  |  | APLPs | 3-4 | mixed | 12 | 12 |
|  | Aβ MSD-ECLIA |  | 3-4 | mixed | 6 | 6 |
|  | Synaptosomes |  | 3-4 | mixed | 6 | 6 |
| **Brain anatomy and neuronal morphology** | IHC |  | 3 | mixed | 4 | 4 |
|  | Stereology |  | 3 | mixed | 5 | 5 |
|  | Sholl analysis |  | 4 | mixed | 6 | 5 |
|  | Spine density |  | 4 | mixed | 6 | 5 |
|  | Spine morphology |  | 4 | mixed | 7 | 6 |
|  | Spine density (Golgi staining, young cohort) |  | 4 | mixed | 5 | 5 |
|  | Spine density (Golgi staining, aged cohort) |  | 15-16 | mixed | 6 | 6 |
| **Electrophysiology** | Extracellular field recordings | LTP | 4-5 | mixed | 6 | 5 |
|  |  | APPsα rescue | 4-5 | mixed | 6 | 6 |
|  | Patch-clamp analysis in OHCs | Synaptic scaling | >DIV18 | mixed | 5* | 4-5* |
| **Behavior** | IntelliCage tests |  | 7-9 | female only | 14 | 17 |
|  | T-maze, radial arm maze, grip strength, rotarod |  | 12-14 | mixed | 13 | 16 |

Experiments indicated with the same color were performed using the same set of animals, whereas black color denotes tests performed with distinct sets of animals. For all experiments except for IntelliCage tests, mice of both sexes (mixed), with balanced numbers of females and males, were used.

Patch-clamp analysis was performed in organotypic entorhino-hippocampal cultures (OHCs) obtained from mice at postnatal day 3-5 and kept for more than 18 days *in vitro* (DIV).

*denotes the number of OHCs obtained from 3-4 mice per genotype

## Supplementary Figure S3. Normal brain morphology of APPΔS622 knockin mice.

**(A)** Representative images of Nissl-stained coronal brain sections (bregma -1.9) displaying the hippocampus and a section of the prefrontal cortex of wild-type (WT) and APPΔS622 (age: 3 months, both sexes). No obvious morphological changes were observed in APPΔS622 mice; scale bar: 500 µm.

**(B, C)** Stereological quantification of hippocampal (B) and cortical (C) volume revealed no significant differences between WT and APPΔS622 mice (age: 3 months, both sexes, N = 5 mice/genotype).

**(D, E)** Double immunohistochemistry of coronal brain sections (40 μm) of WT and APPΔS622 animals stained for Ctip2 (green, expressed in layers V/VI) and calbindin (red, expressed in layers II/III and partially in layer V). Cortical lamination and layer thickness appeared comparable between genotypes. Scale bar: 100 μm. **(E)** In the violin plots, the dashed line represents the median, while the pointed lines indicate the quartiles.

**(F, G)** Assessment of astrogliosis using NeuN (neuronal marker, green) and GFAP (astrocyte marker, red) with DAPI nuclear counterstaining (blue). **(G)** Quantification of GFAP⁺ astrocytes normalized to DAPI⁺ cells showed no significant difference between WT and APPΔS622 mice. Scale bar: 400 µm.

**(H, I)** Analysis of microgliosis using the neuronal marker NeuN (green) and the microglia marker Iba1 (red) with DAPI counterstaining (blue). **(I)** Quantification revealed no significant change in microglial cell numbers between genotypes**.** Scale bar: 400 µm.

Data information for F-I: n = 12 coronal brain sections from N = 4 mice/genotype (age: 3 months, both sexes). Data were analyzed using an unpaired Student’s *t*-test and are shown as mean ± SEM. ns, not significant.

Supplementary Figure S4. Sholl analysis reveals unchanged dendritic complexity of CA1 pyramidal neurons in APPΔS622 mice.

**(A)** Representative 3D reconstructions of CA1 pyramidal neurons from WT (left) and APPΔS622 (right) mice. Schematic illustration of the Sholl concentric spheres placed around the soma of a reconstructed CA1 pyramidal neuron to assess dendritic complexity.

**(B)** Sholl analysis of basal dendrites showing the number of intersections plotted against the distance from the soma revealed no significant differences between WT and APPΔS622 neurons.

**(C)** Total number of primary dendrites was not significantly changed in APPΔS622 neurons compared to WT.

**(D)** Total basal dendritic length was comparable between genotypes.

**(E)** Total number of basal dendritic intersections was not altered in APPΔS622 neurons compared to WT.

**(F)** The number of dendritic nodes in basal dendrites was unchanged between the two genotypes.

**(G)** Sholl analysis of apical dendrites showing the number of intersections as a function of distance from the soma revealed no differences between WT and APPΔS622 neurons.

**(H)** Total apical dendritic length was similar between genotypes.

**(I)** Total number of apical dendritic intersections was not altered in APPΔS622 neurons.

**(J)** The number of dendritic nodes in apical dendrites was comparable between WT and APPΔS622 mice.

Data information: n = number of neurons; N = number of animals (age: 3–4 months, both sexes). Data were analyzed using an unpaired Student’s *t*-test and, for multiple group comparisons, using two-way ANOVA with Bonferroni's multiple comparisons *post hoc* test. Data are presented as mean ± SEM. ns not significant.

Supplementary Figure S5. IntelliCage setup and behavioral testing paradigm.

**(1)** Schematic overview of the IntelliCage apparatus. The IntelliCage consists of four operant conditioning chambers located in the corners of a large home cage, allowing automated monitoring of group-housed, radio-frequency identification (RFID)-tagged mice. Female mice (mixed genotypes) were housed under an inverted 12:12 h light–dark cycle, and their behavior (corner visits, nose pokes, and licks) was recorded automatically. Nose pokes were detected by infrared beams and controlled access to water bottles via motorized doors. Triangular shelters placed in the center served as resting areas and platforms for food access. Behavioral protocols were controlled by IntelliCage Plus software.

**(2)** Free adaptation phase (days 1–4) during which all doors remained open to allow mice to habituate to the cage and learn to obtain water in the corners.

**(3)** Nose poke adaptation phase (3 days) in which all doors were initially closed and the first nose poke during each visit opened the door for 5 s to allow drinking. Performance on day 3 served as baseline for subsequent tasks.

**(4)** Serial reaction time task. The first nose poke during a visit determined the correct side and initiated a delay period, after which three green LEDs illuminated, and the door opened for 5 s to allow drinking. Nose pokes during the delay were counted as premature responses. The task included a 3-day baseline phase (0 s delay), followed by randomized delays (0.5–2.5 s) with 4 training days without consequences for premature responses.

**(5)** Nose poke adaptation was repeated for 3 days as described in (3).

**(6)** Adaptation to drinking sessions (4 days), during which water was available only during four 1 h sessions per day.

**(7)** Corner preference task. Each mouse was assigned one rewarded corner where nose pokes opened the door during drinking sessions (acquisition, 4 days).

**(8)** Adaptation to drinking sessions was repeated for 4 days as described in (6).

**(9)** Chaining task. The rewarded corner depended on the animal’s previous visit, following a fixed clockwise or counterclockwise sequence. Mice were trained for 6 days in one direction.

## Supplementary Figure S6. Normal motor strength and coordination in APPΔS622 mice.

**(A)** Forelimb grip strength measured as maximal force (g) was comparable between aged WT and APPΔS622 mice, indicating no impairment in muscular strength.

**(B)** Motor coordination and balance assessed using an accelerating rotarod revealed no significant difference in latency to fall between genotypes.

Data were analyzed using two-way ANOVA with Bonferroni's multiple comparisons *post hoc* test. Data information: N = 15-17 mice of both sexes/genotype (age: 13-14 months). Data are presented as mean ± SEM.

# Supplementary References

1.	Amrein I, Nosswitz M, Slomianka L, van Dijk RM, Engler S, Klaus F, et al. Septo-temporal distribution and lineage progression of hippocampal neurogenesis in a primate (Callithrix jacchus) in comparison to mice. Front Neuroanat. 2015 Jun 29;9. doi:10.3389/fnana.2015.00085

2.	Gundersen HJG, Jensen EBV, Kiêu K, Nielsen J. The efficiency of systematic sampling in stereology — reconsidered. Journal of Microscopy. 1999;193(3):199–211. doi:10.1046/j.1365-2818.1999.00457.x

3.	Slomianka L, West MJ. Estimators of the precision of stereological estimates: An example based on the CA1 pyramidal cell layer of rats. Neuroscience. 2005 Jan;136(3):757–67. doi:10.1016/j.neuroscience.2005.06.086

4.	Ring S, Weyer SW, Kilian SB, Waldron E, Pietrzik CU, Filippov MA, et al. The Secreted β-Amyloid Precursor Protein Ectodomain APPsα Is Sufficient to Rescue the Anatomical, Behavioral, and Electrophysiological Abnormalities of APP-Deficient Mice. J Neurosci. 2007 Jul 18;27(29):7817–26. doi:10.1523/JNEUROSCI.1026-07.2007 PubMed PMID: 17634375.

5.	Koentgen F, Lin J, Katidou M, Chang I, Khan M, Watts J, et al. Exclusive transmission of the embryonic stem cell-derived genome through the mouse germline. genesis. 2016 Jun 1;54(6):326–33. doi:10.1002/dvg.22938
